# Supplementary material for: Synthesis and Properties of New 3-Heterylamino-Substituted 9-Nitrobenzanthrone Derivatives
Source: Molecules. 2023 Jul 2;28(13):5171. doi: 10.3390/molecules28135171 (PMC10343498; doi:10.3390/molecules28135171)
Supplement: Supplementary file 1 [file molecules-28-05171-s001.zip › molecules-2466865-supplementary.pdf]

Supplementary Material

Synthesis and Properties of New 3-Heterylamino-Substituted 9-Nitrobenzanthrone Derivatives

Armands Maļeckis [1], Marija Cvetinska [1], Aleksandrs Puckins [2], Sergejs Osipovs [2], Jelizaveta Sirokova [2], Sergey Belyakov [3] and Elena Kirilova [2,*]

1 Institute of Technology of Organic Chemistry, Faculty of Materials Science and Applied Chemistry, Riga Technical University, P. Valdena Str. 3, LV-1048 Riga, Latvia
2 Department of Applied Chemistry, Institute of Life Sciences and Technology, Daugavpils University, LV-5401 Daugavpils, Latvia
3 Latvian Institute of Organic Synthesis, Aizkraukles Str. 21, LV-1006 Riga, Latvia; serg@osi.lv
* Correspondence: jelena.kirilova@du.lv

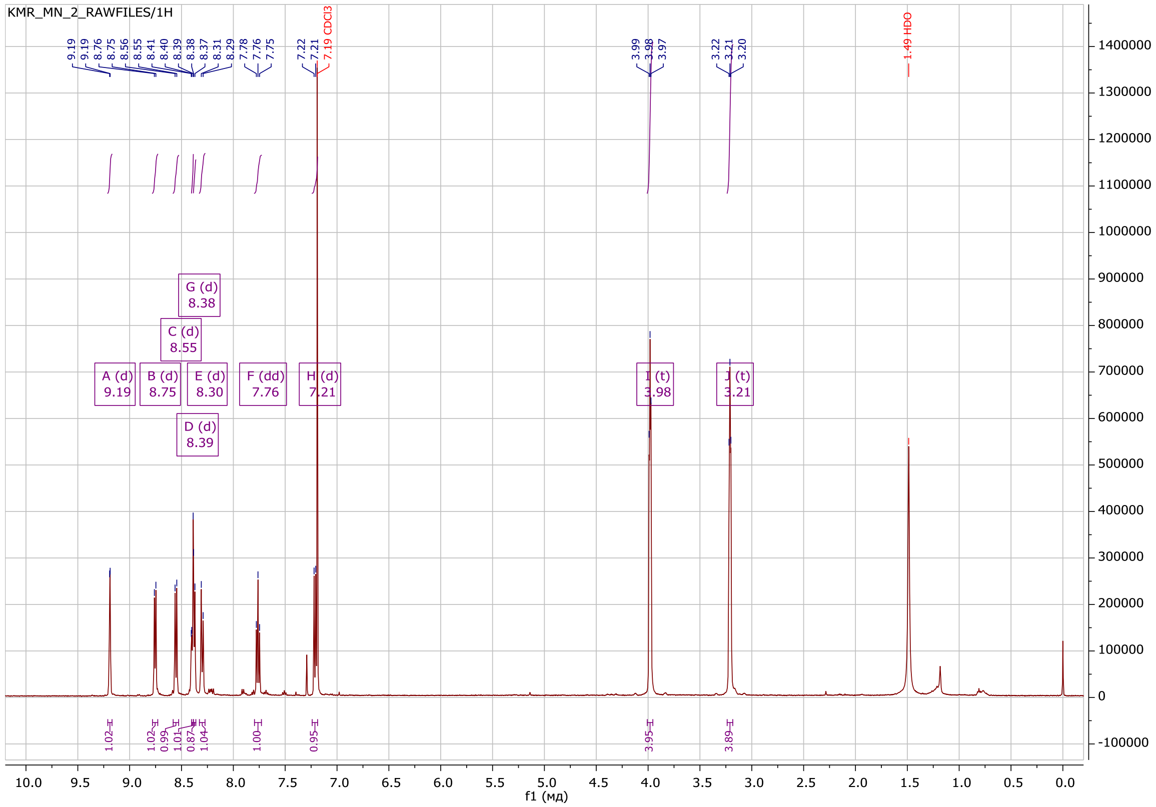

Figure S1. ^{1}H-NMR (500 MHz, $CDCl_3$) spectrum of compound **2.**

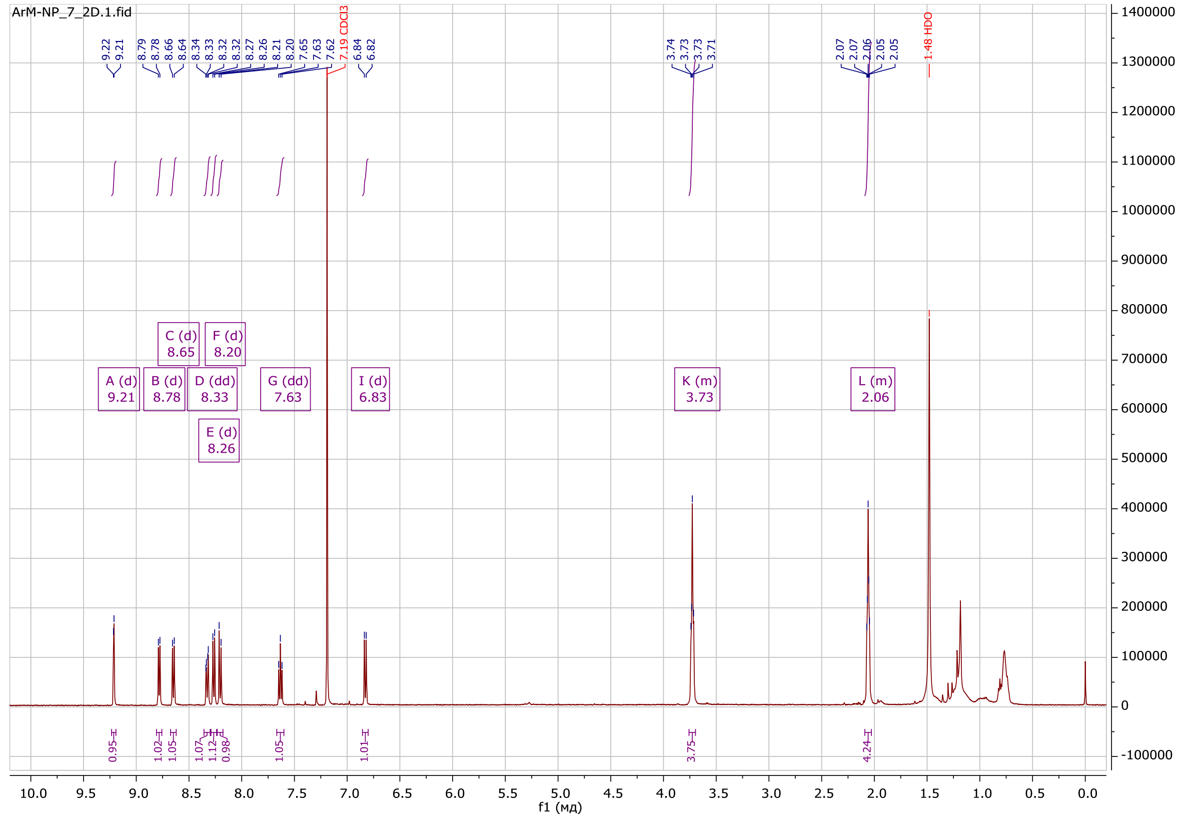

Figure S2. ^{1}H-NMR (500 MHz, $CDCl_3$) spectrum of compound **3.**

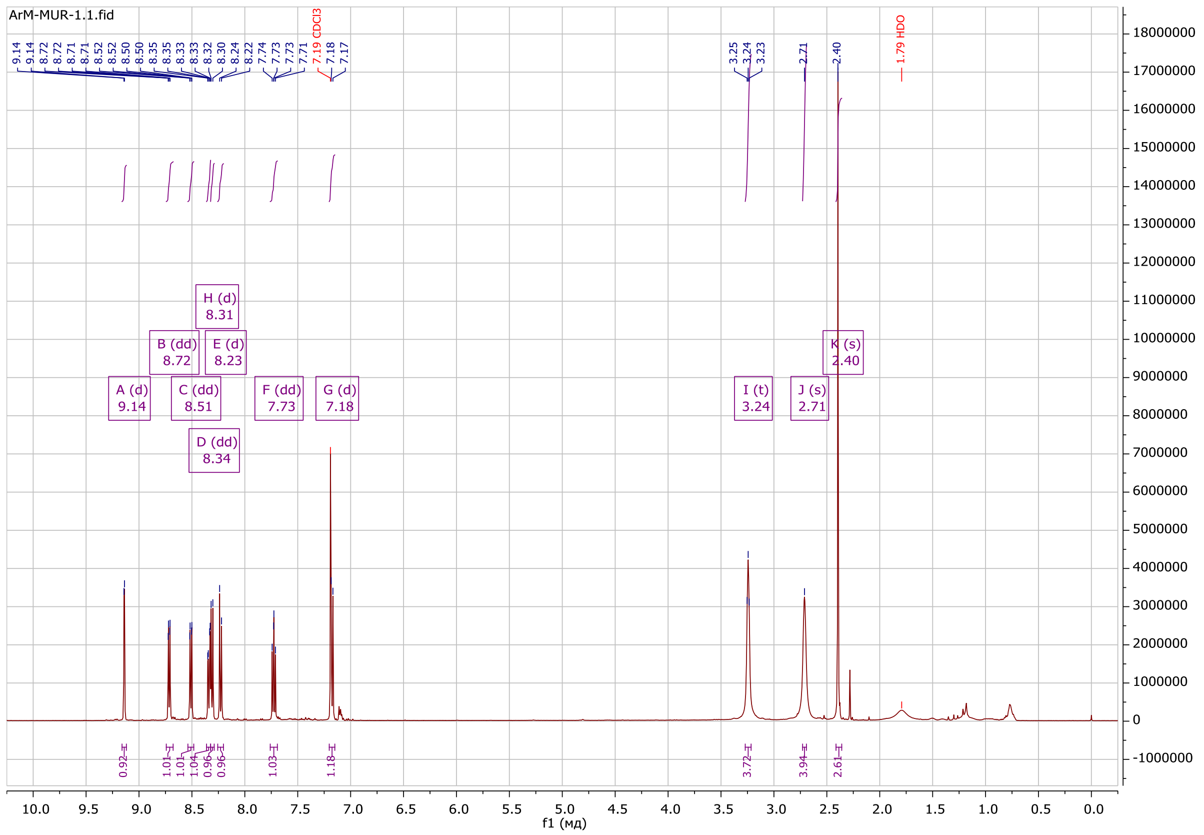

Figure S3. ^{1}H-NMR (500 MHz, $CDCl_3$) spectrum of compound **4.**

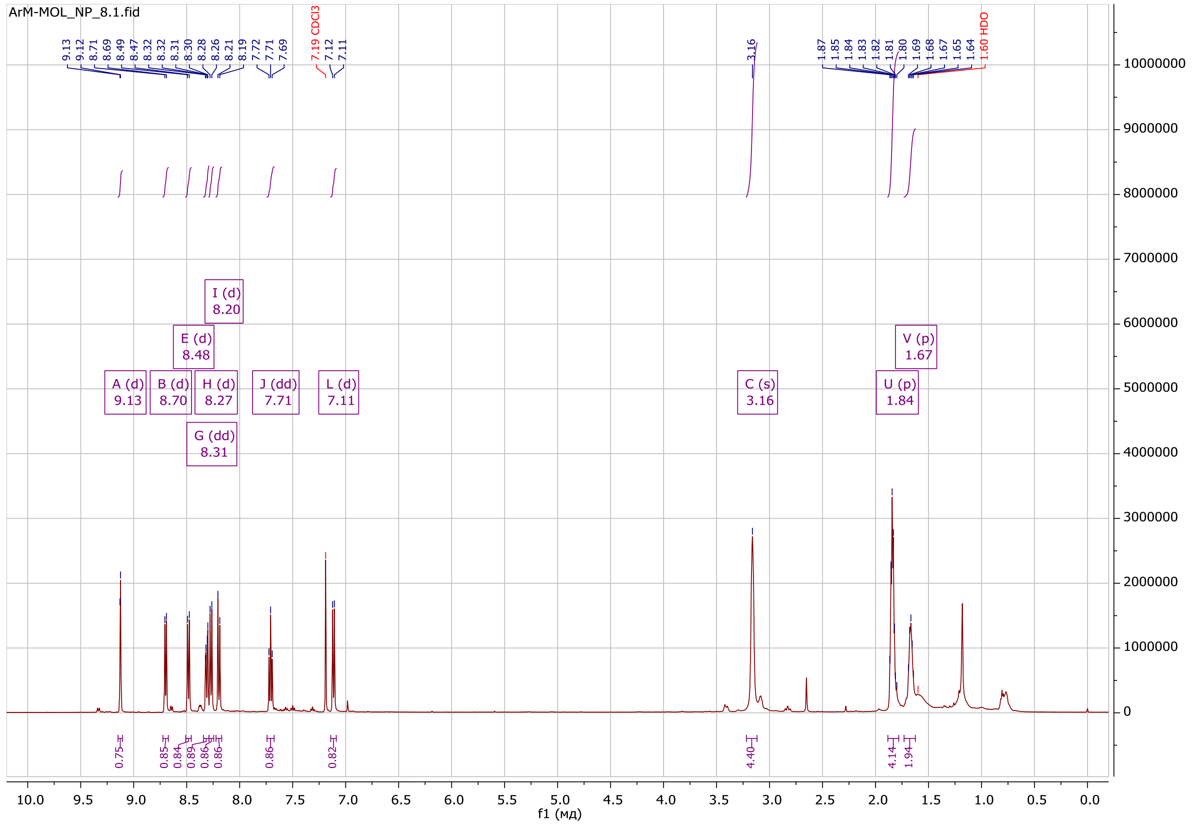

Figure S4. ^{1}H-NMR (500 MHz, $CDCl_3$) spectrum of compound **5.**

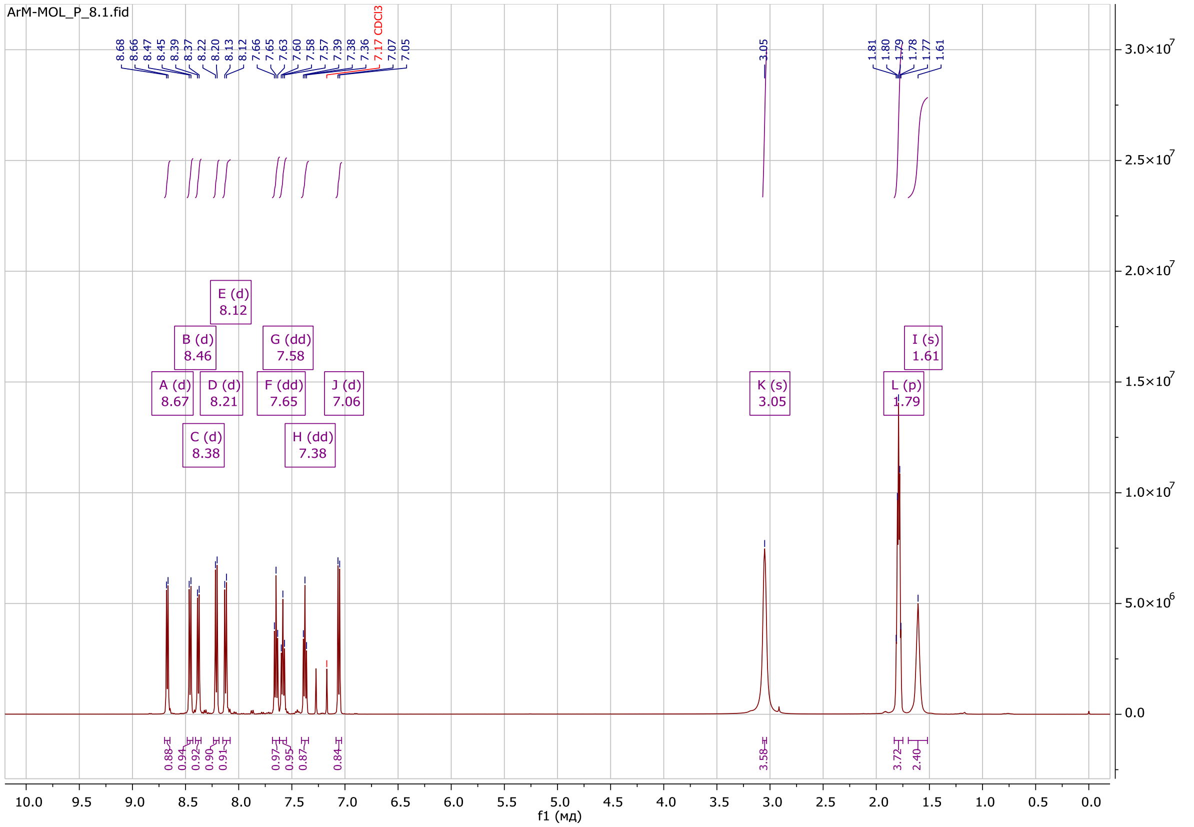

Figure S5. ^{1}H-NMR (500 MHz, $CDCl_3$) spectrum of compound **6.**

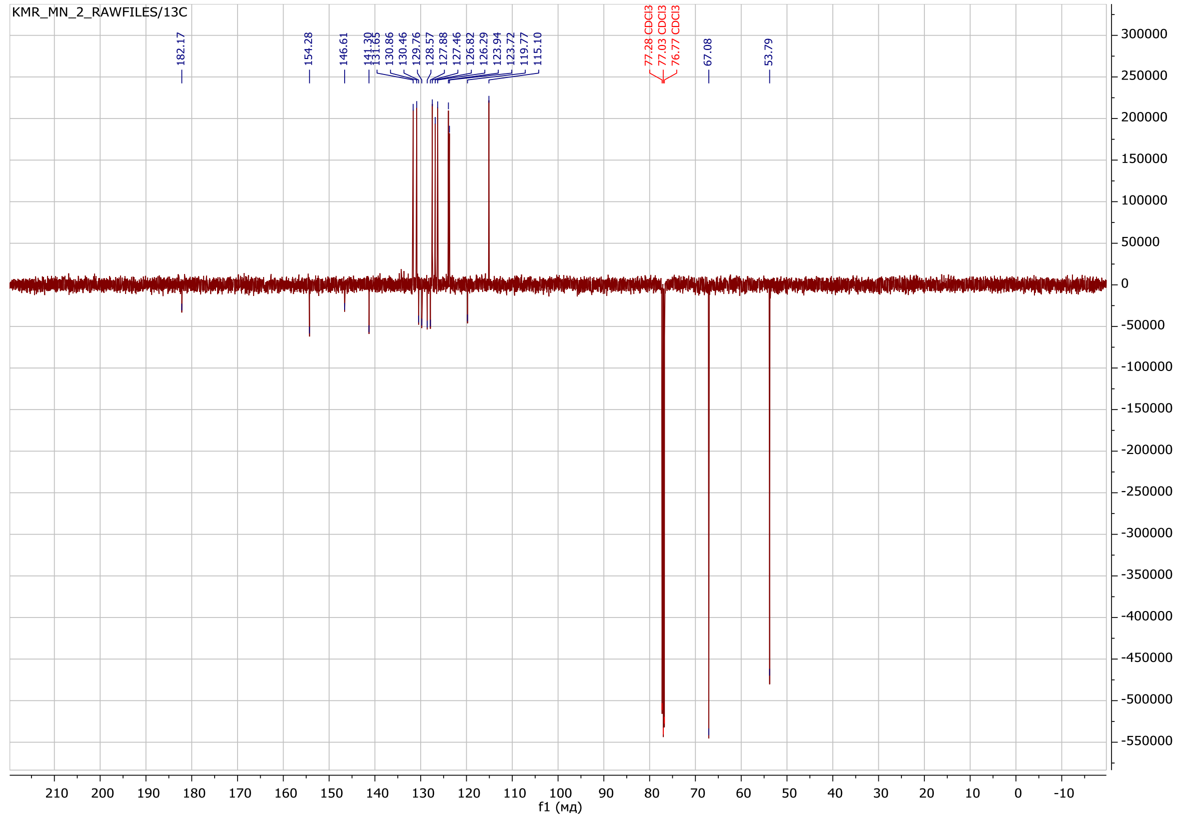

Figure S6. APT NMR (126 MHz, $CDCl_3$) spectrum of compound **2.**

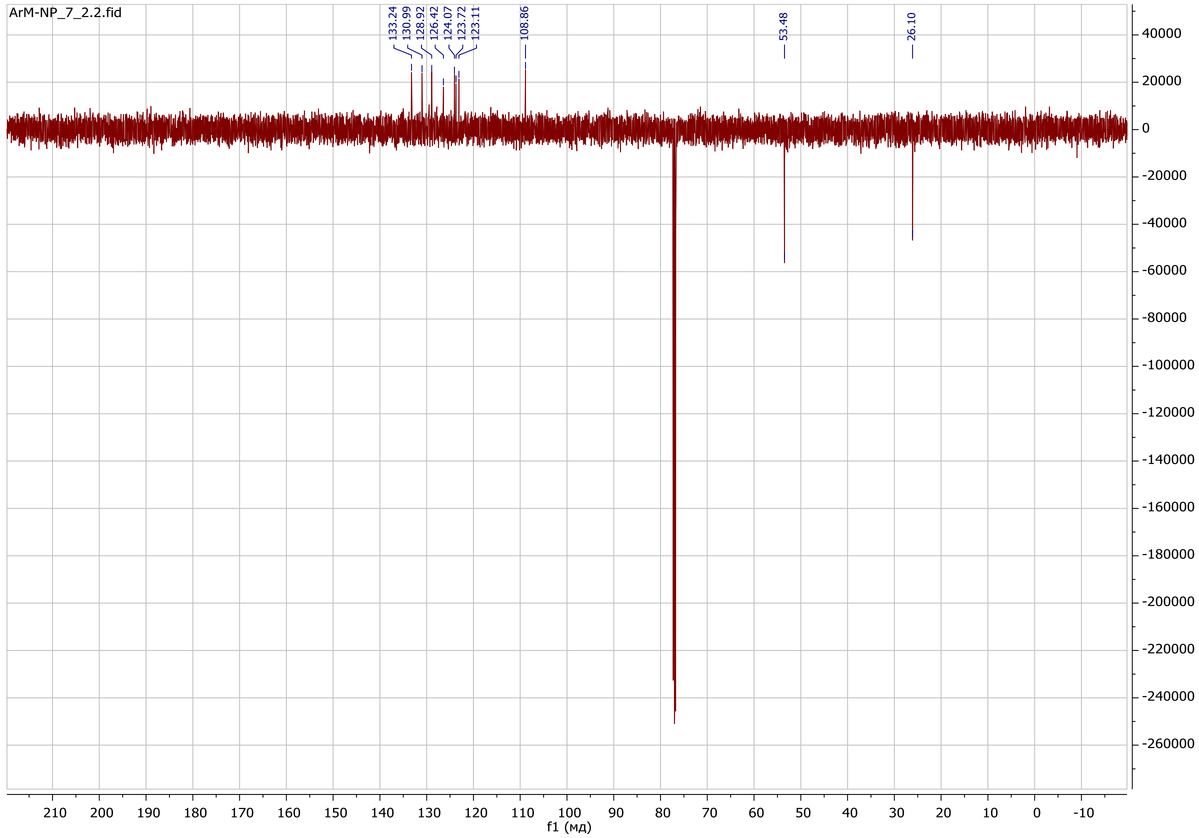

Figure S7. APT NMR (126 MHz, $CDCl_3$) spectrum of compound **3.**

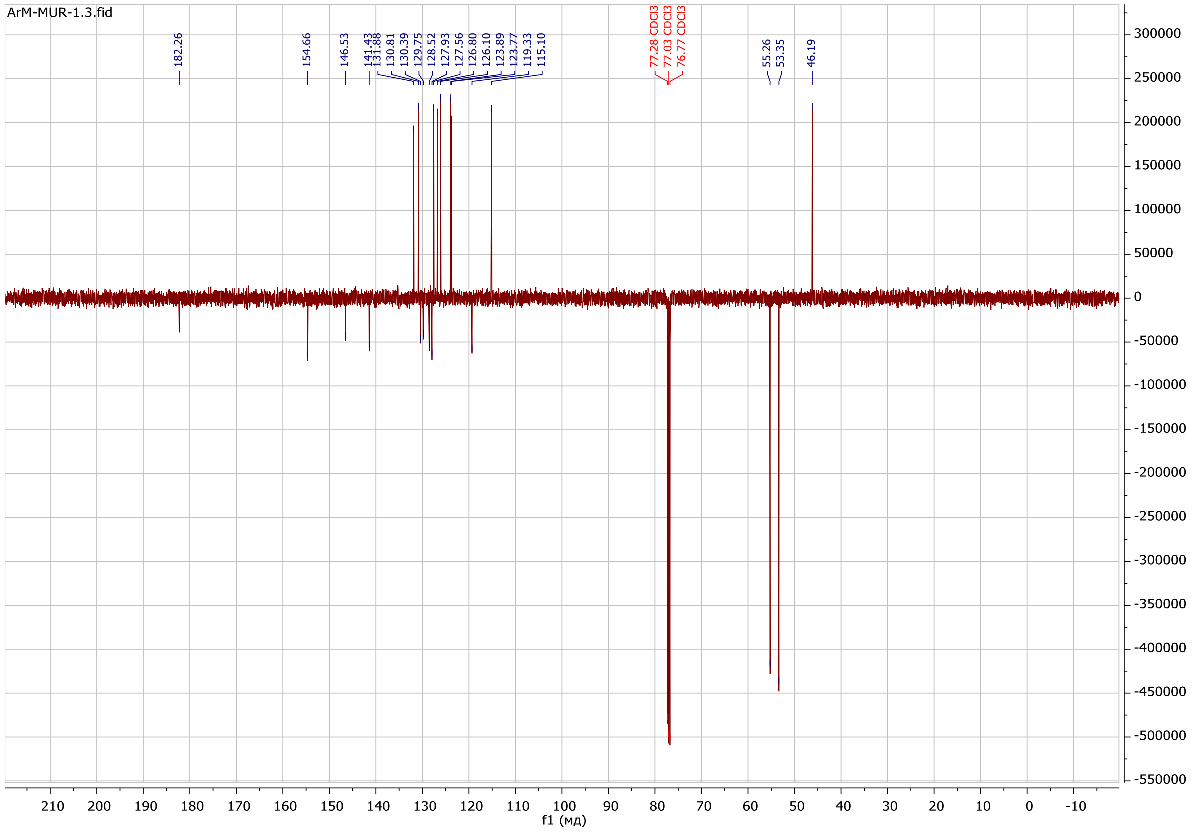

Figure S8. APT NMR (126 MHz, $CDCl_3$) spectrum of compound **4.**

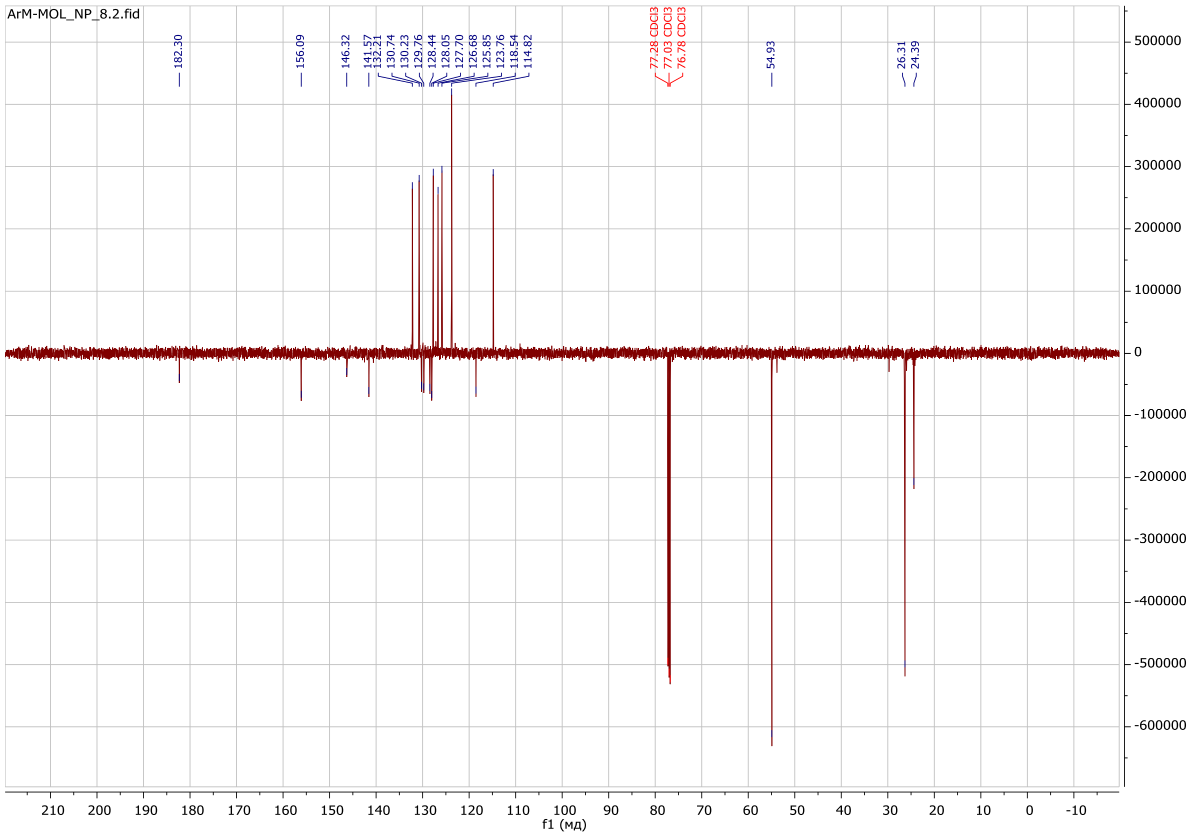

Figure S9. APT NMR (126 MHz, $CDCl_3$) spectrum of compound **5.**

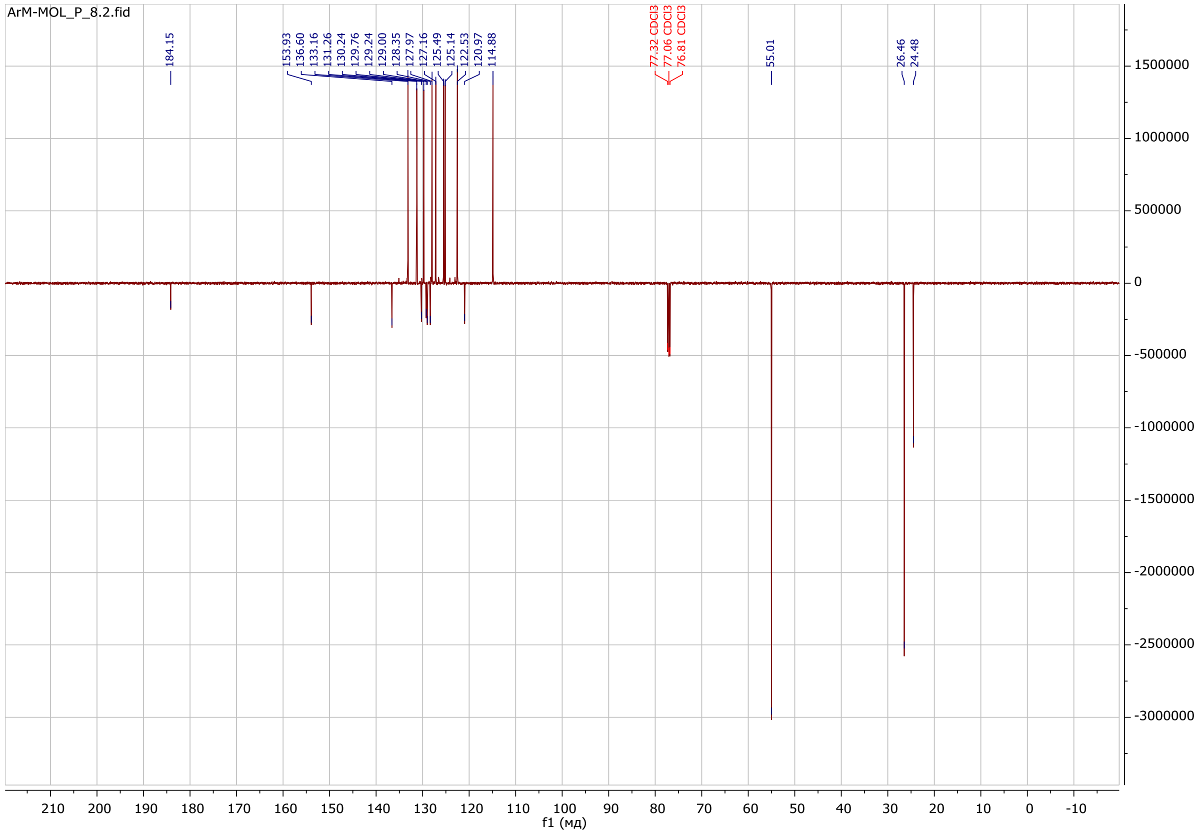

Figure S10. APT NMR (126 MHz, $CDCl_3$) spectrum of compound **6.**

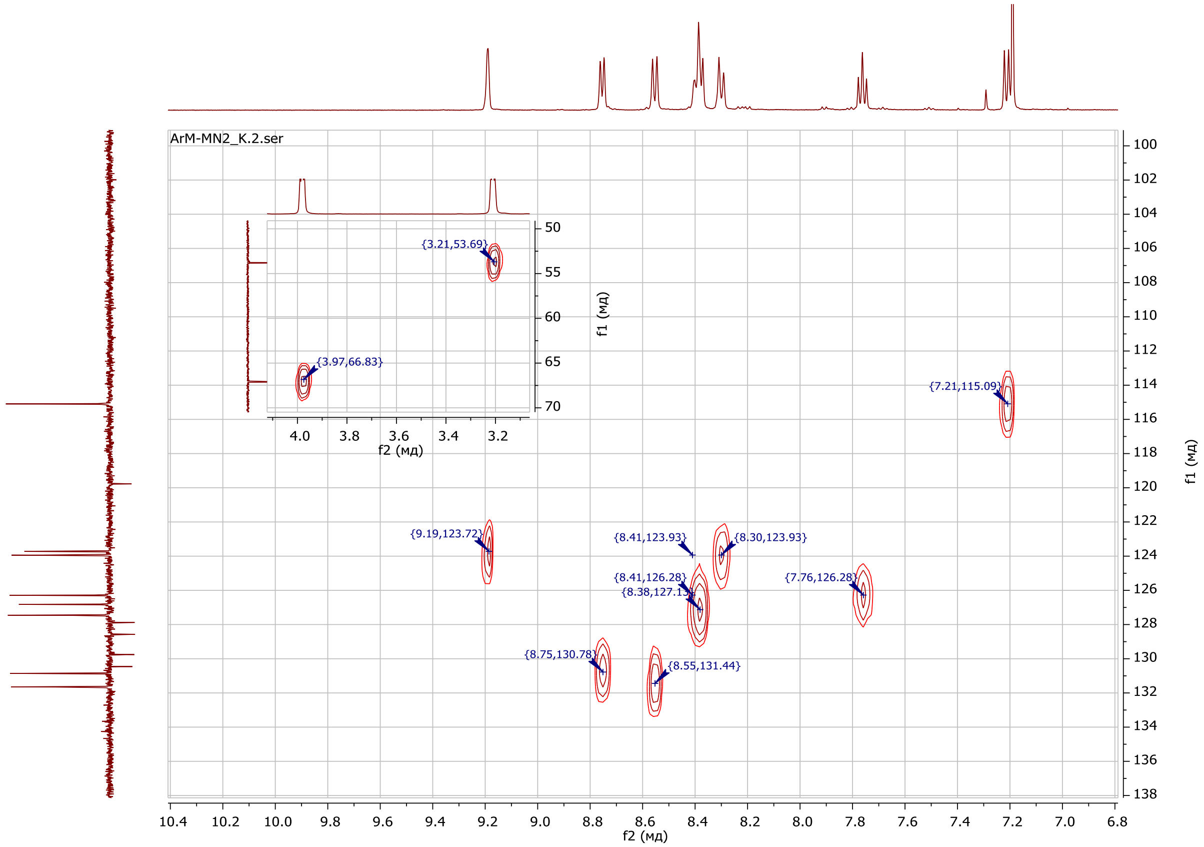

Figure S11. Expanded HSQC NMR spectrum ($CDCl_3$) of compound **2.**

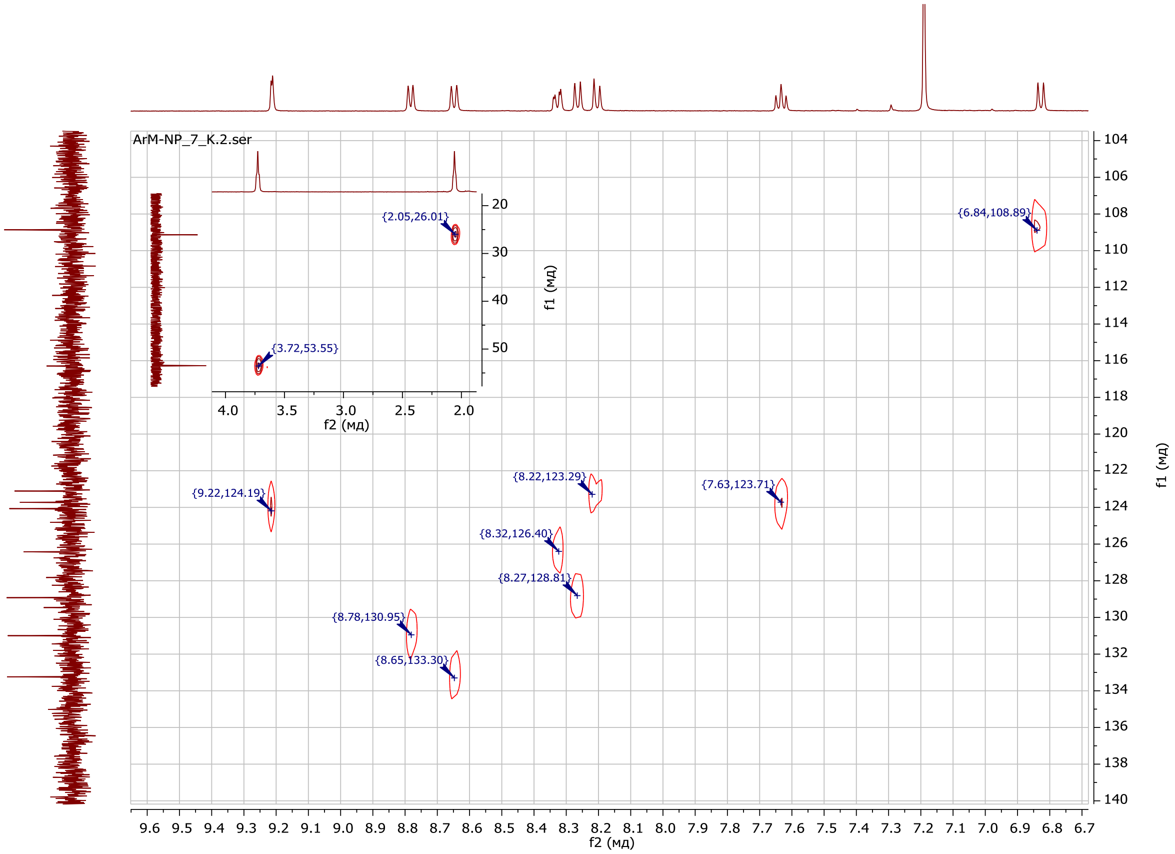

Figure S12. Expanded HSQC NMR spectrum ($CDCl_3$) of compound **3.**

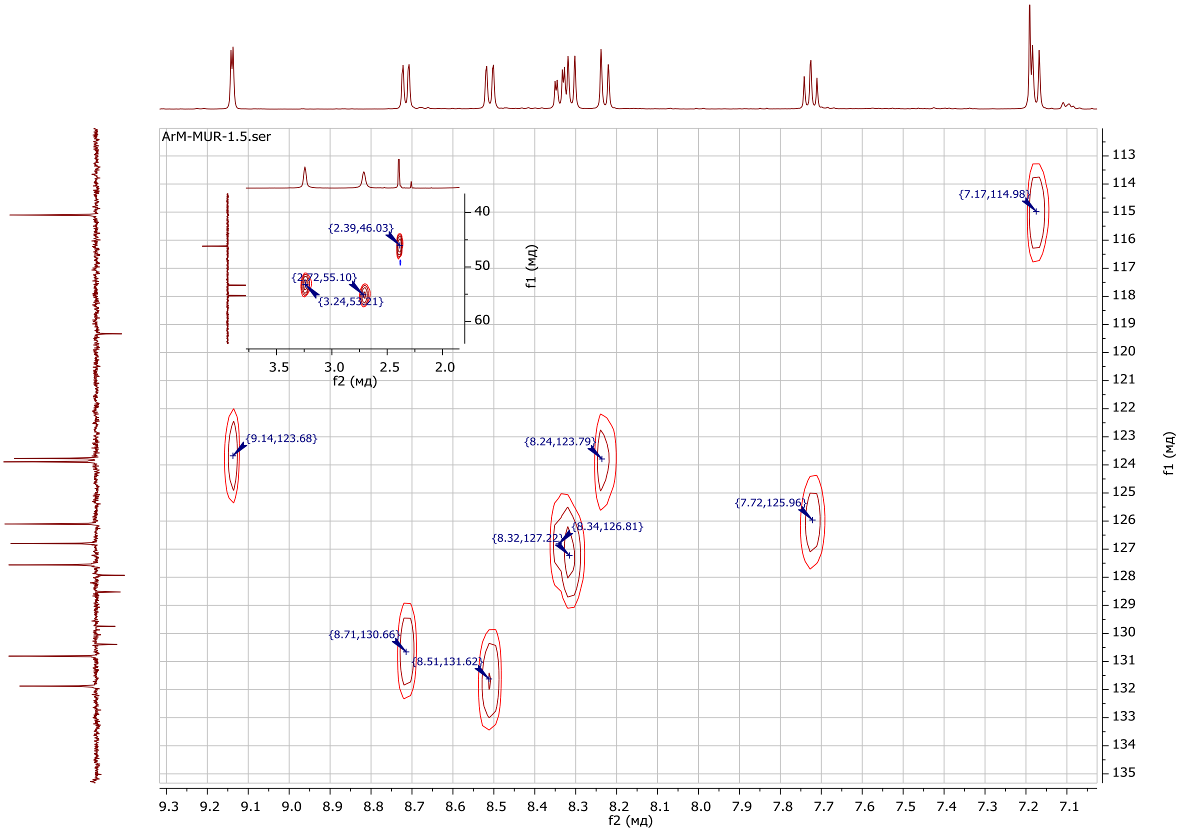

Figure S13. Expanded HSQC NMR spectrum ($CDCl_3$) of compound **4.**

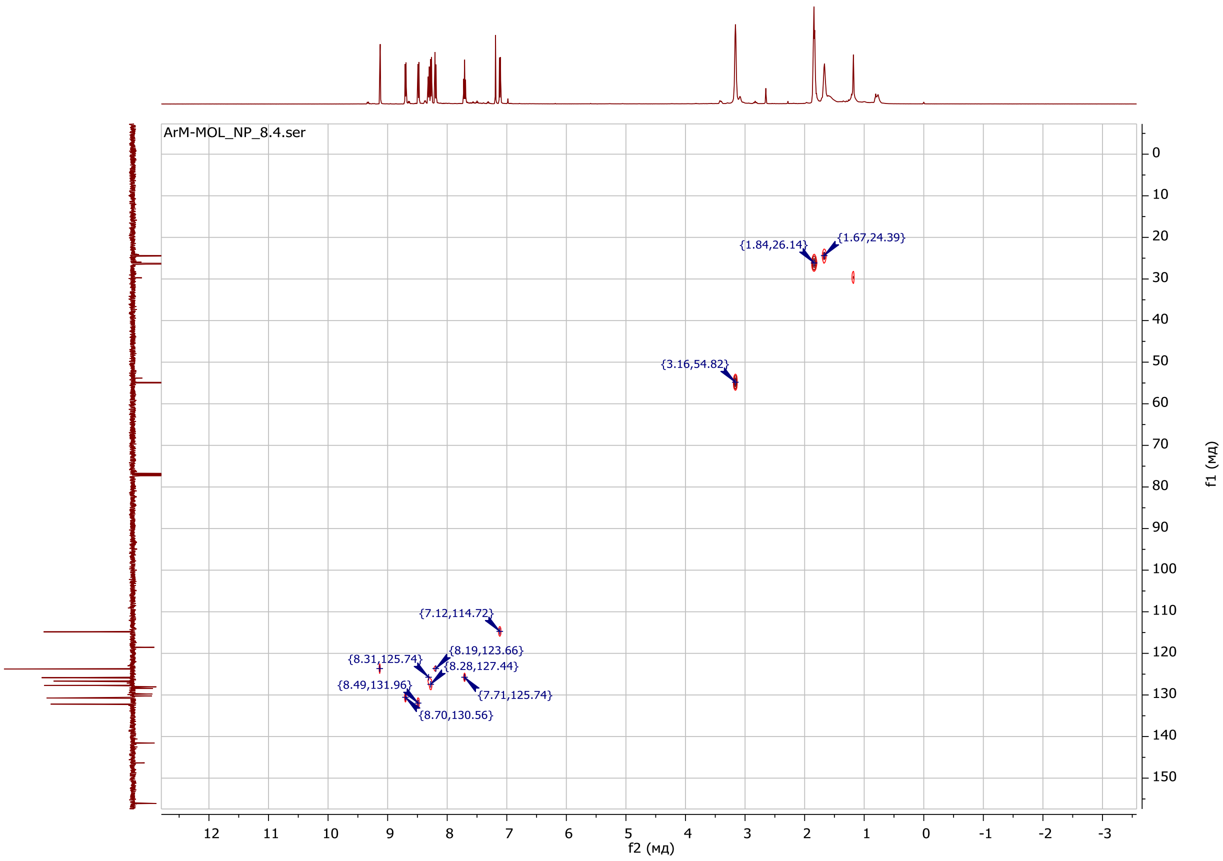

Figure S14. HSQC NMR spectrum ($CDCl_3$) of compound **5.**

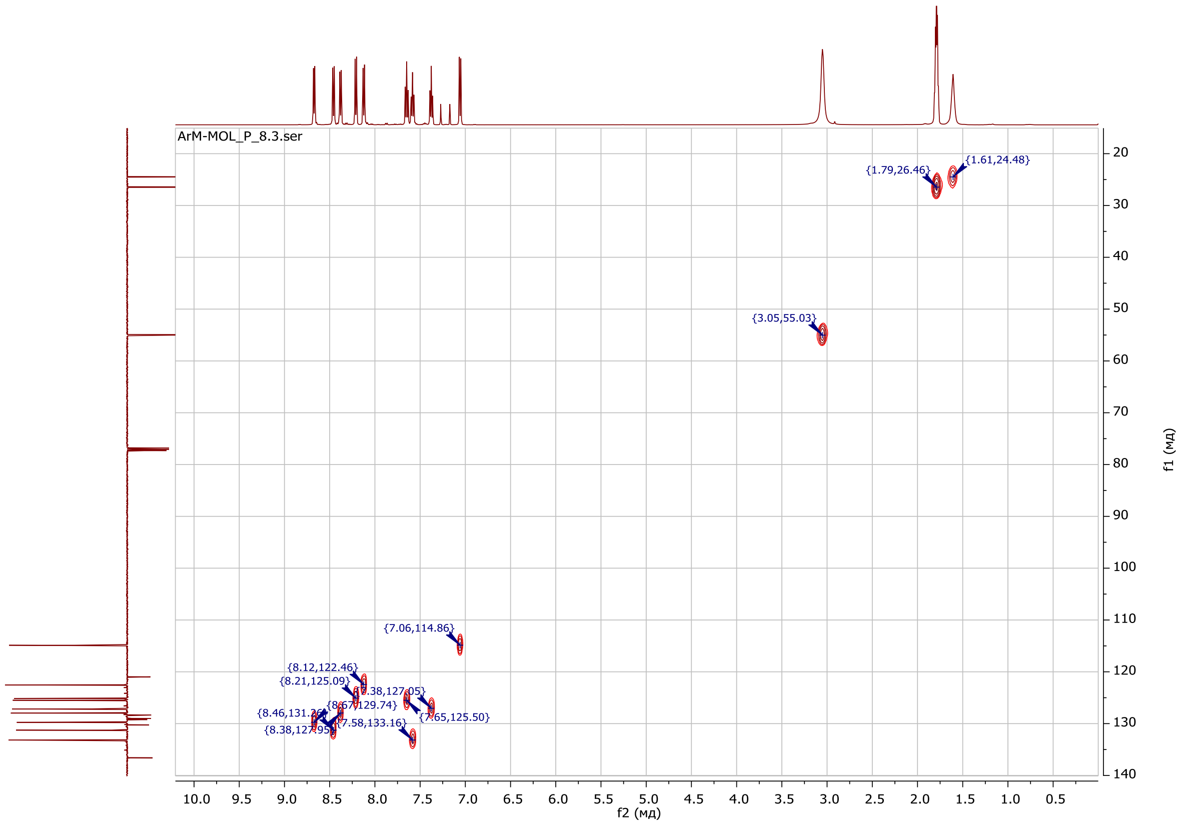

Figure S15. HSQC NMR spectrum ($CDCl_3$) of compound **6.**

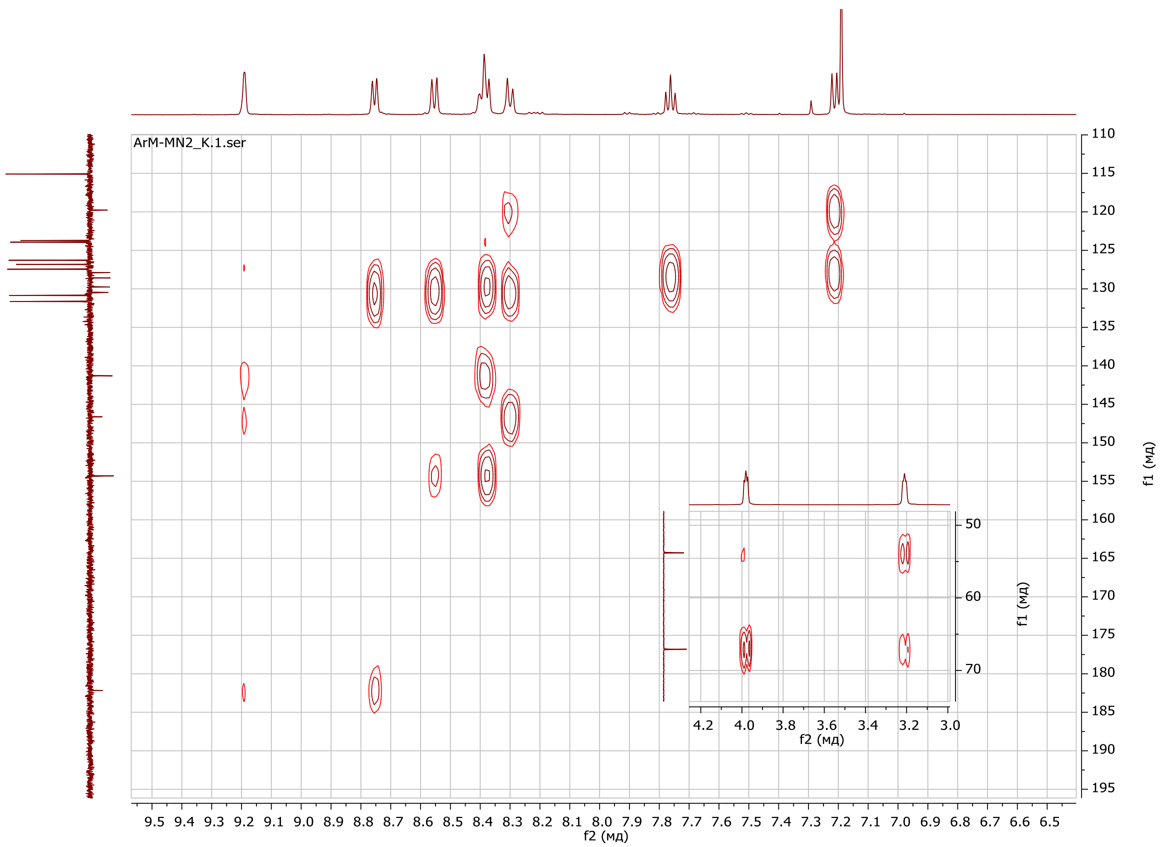

Figure S16. Expanded HMBC spectrum ($CDCl_3$) of compound **2.**

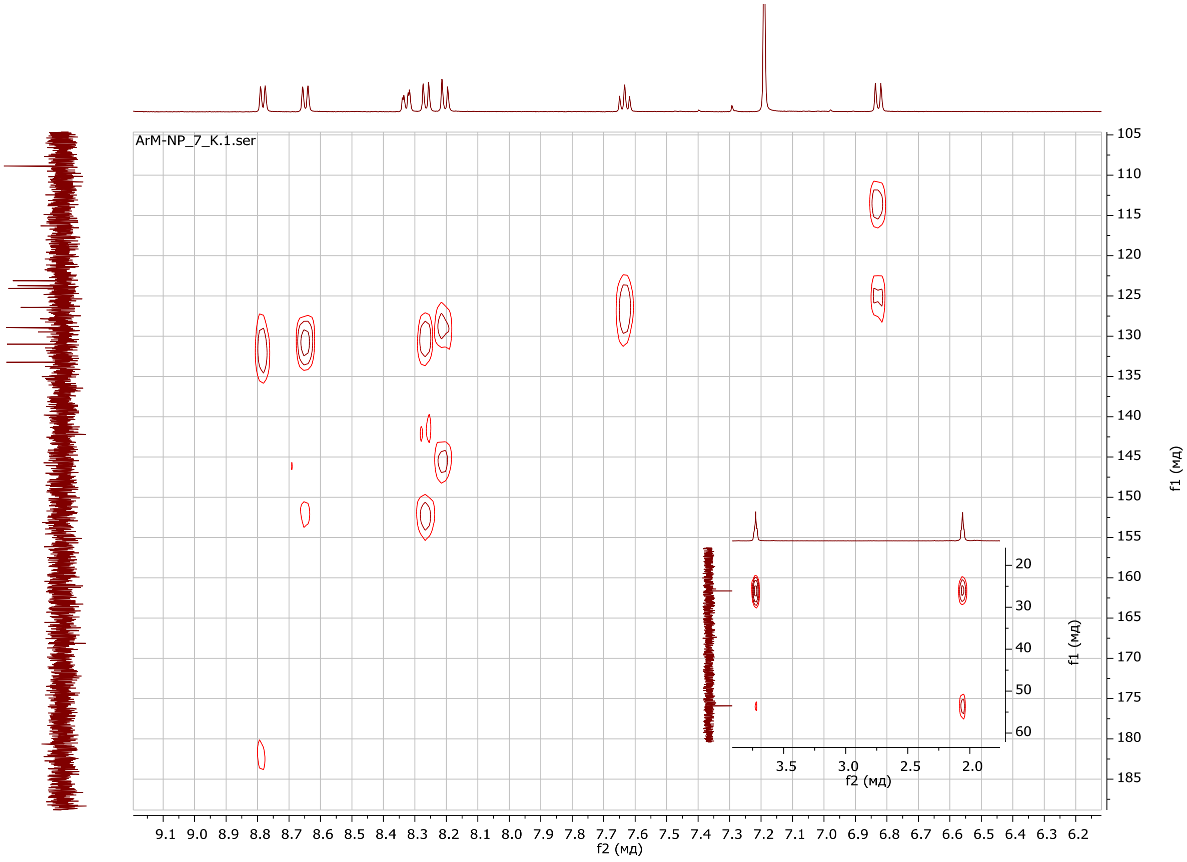

Figure S17. Expanded HMBC spectrum ($CDCl_3$) of compound **3.**

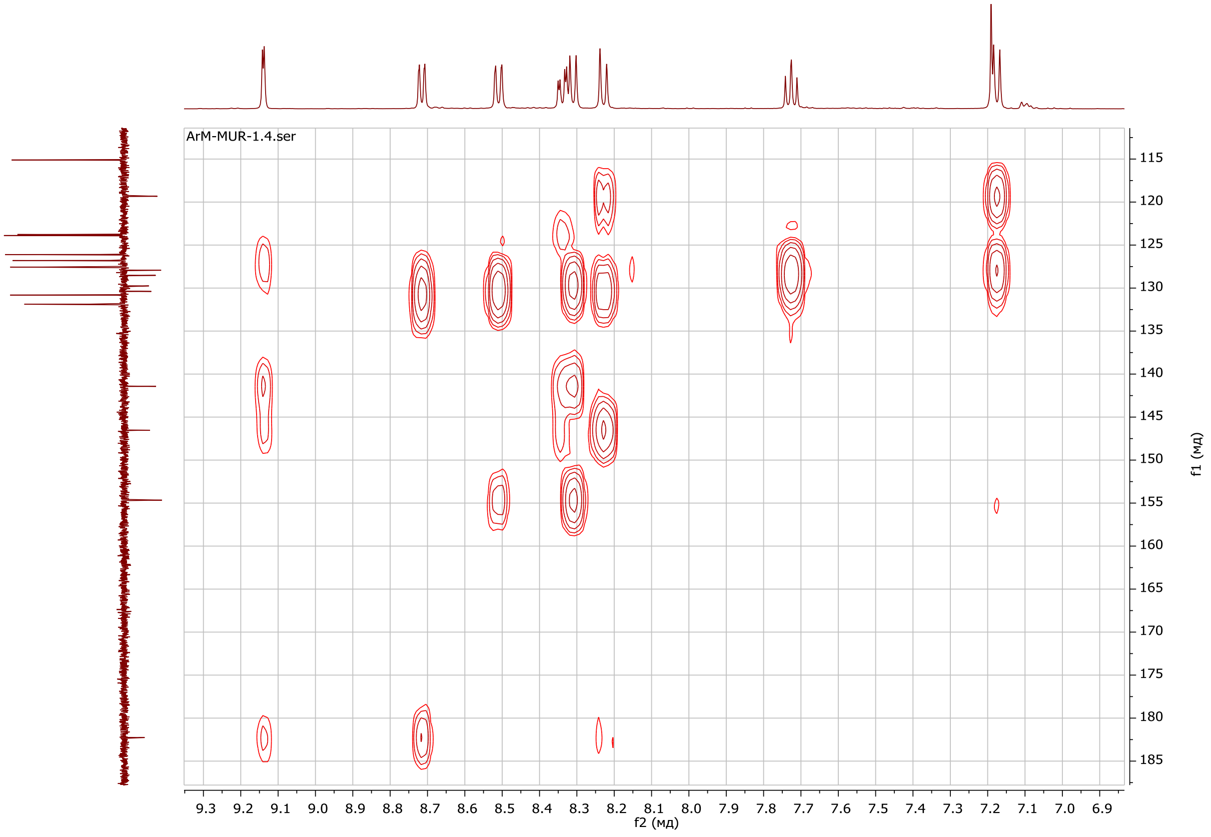

Figure S18. Expanded HMBC spectrum ($CDCl_3$) of compound **4.**

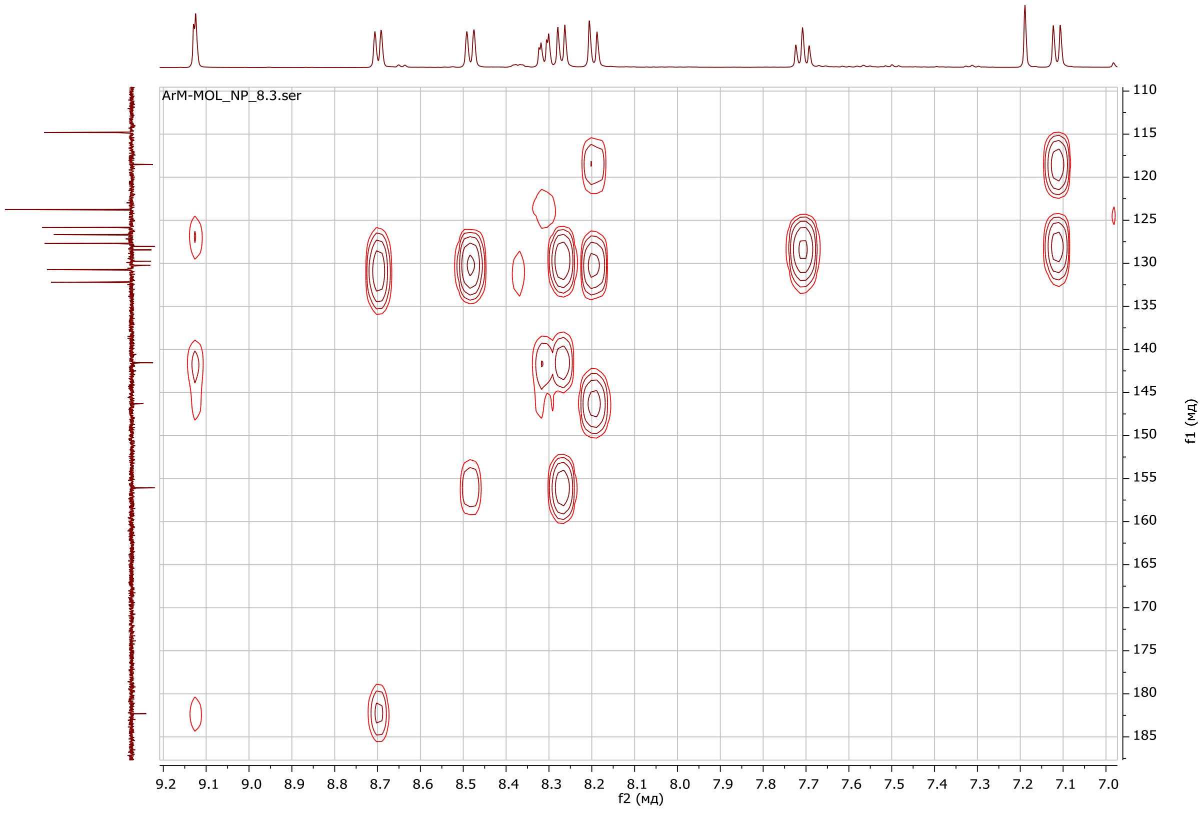

Figure S19. Expanded HMBC spectrum ($CDCl_3$) of compound **5.**

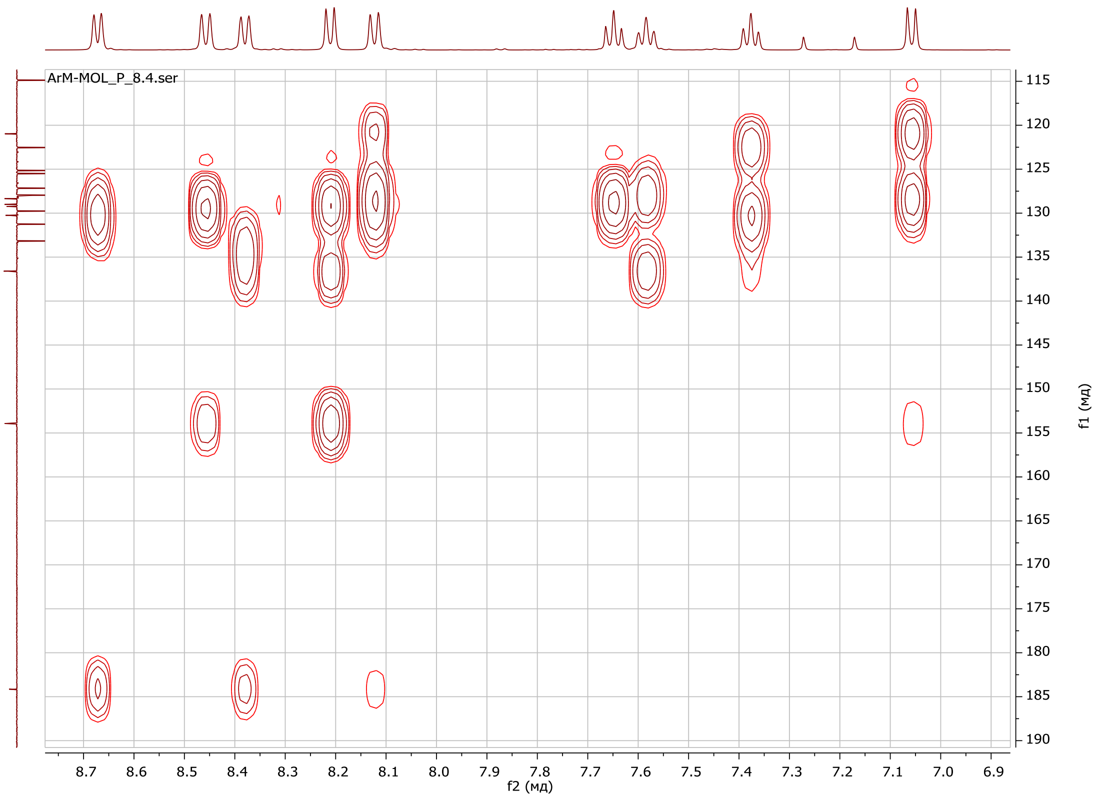

Figure S20. Expanded HMBC spectrum ($CDCl_3$) of compound **6.**

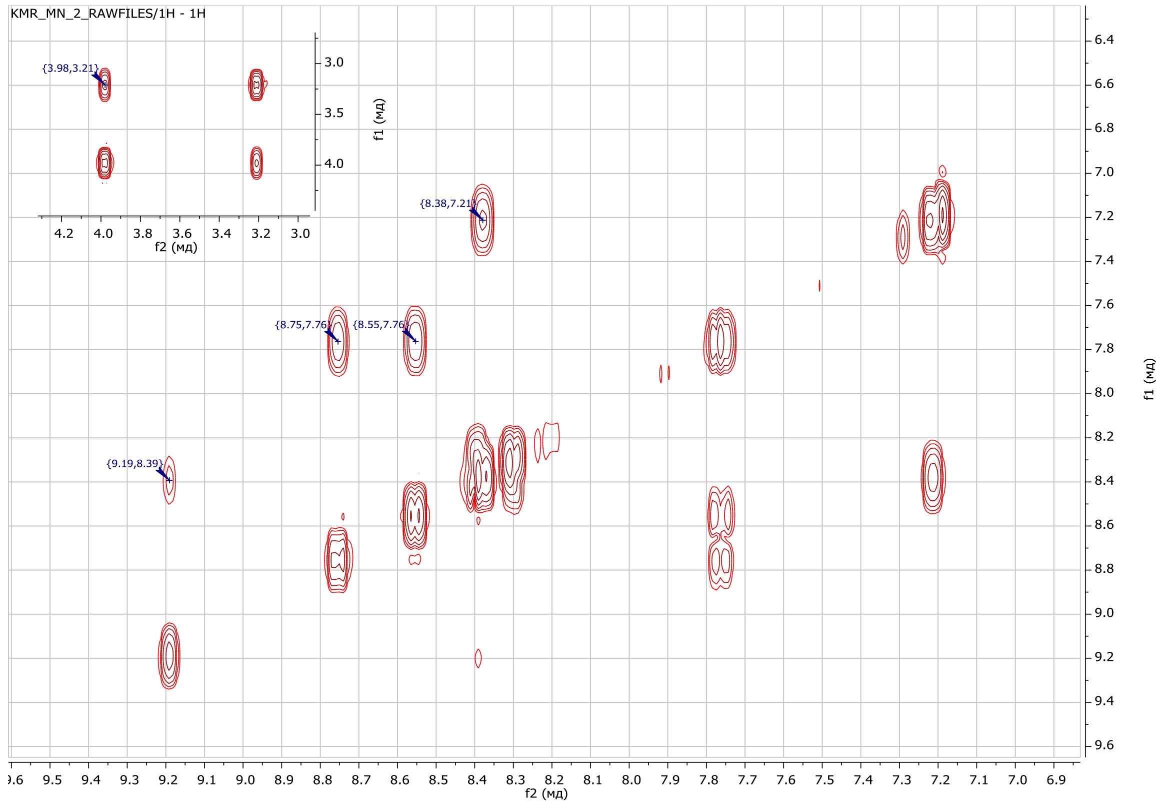

Figure S21. Expanded ^1H-^1H COSY spectrum ($CDCl_3$) of compound **2.**

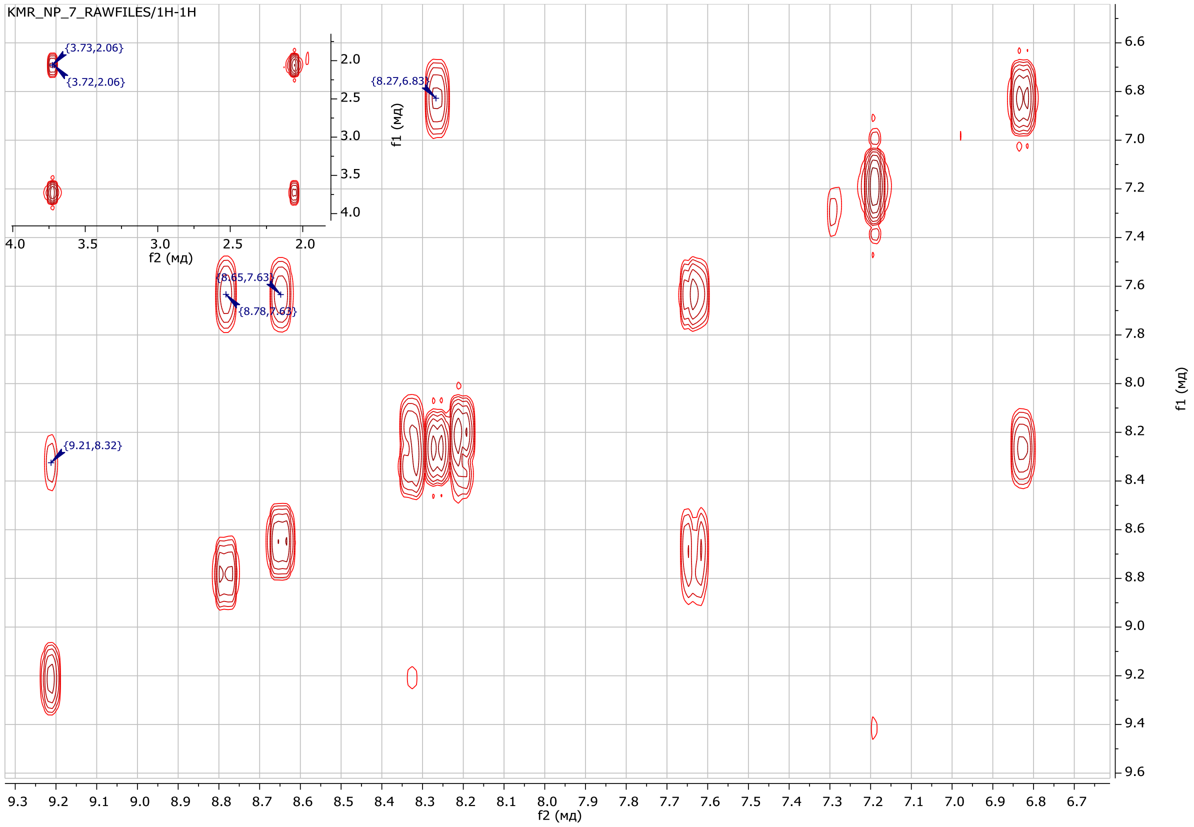

Figure S22. Expanded ^{1}H-^{1}H COSY spectrum ($CDCl_3$) of compound **3**.

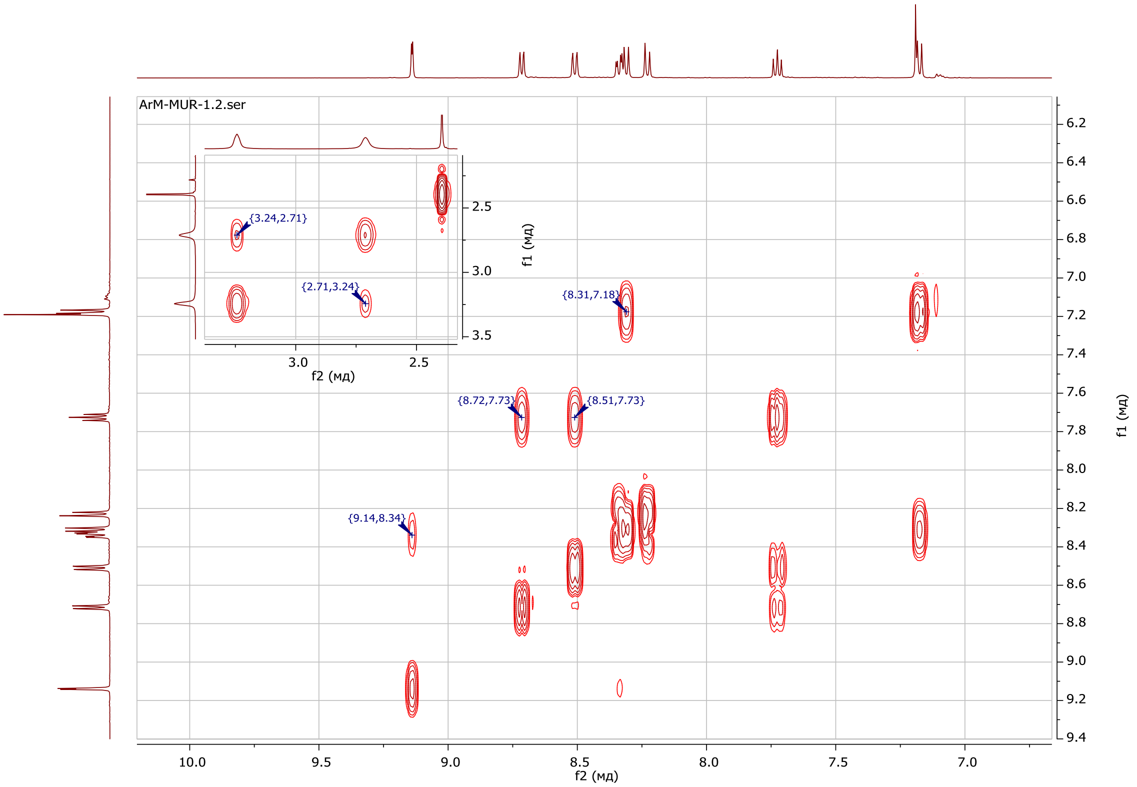

Figure S23. Expanded ^{1}H-^{1}H COSY spectrum ($CDCl_3$) of compound **4**.

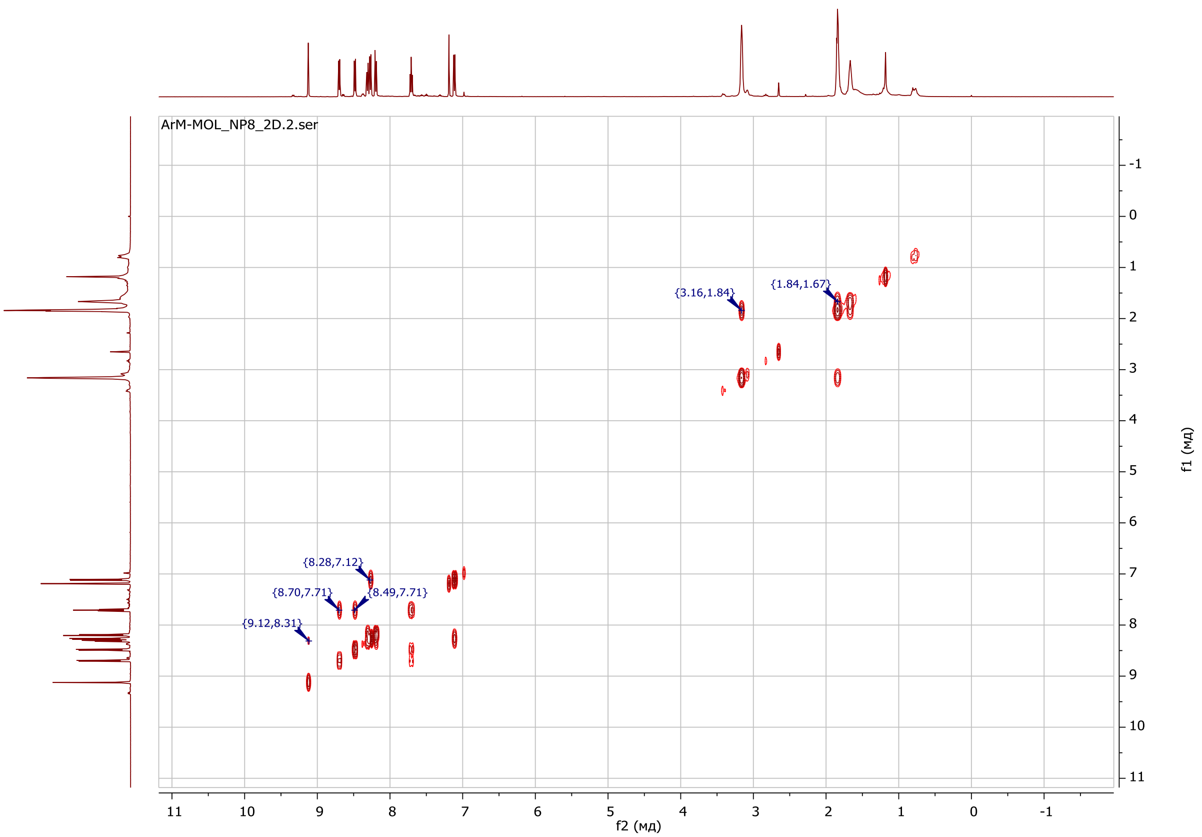

Figure S24. ^{1}H-^{1}H COSY spectrum ($CDCl_3$) of compound **5**.

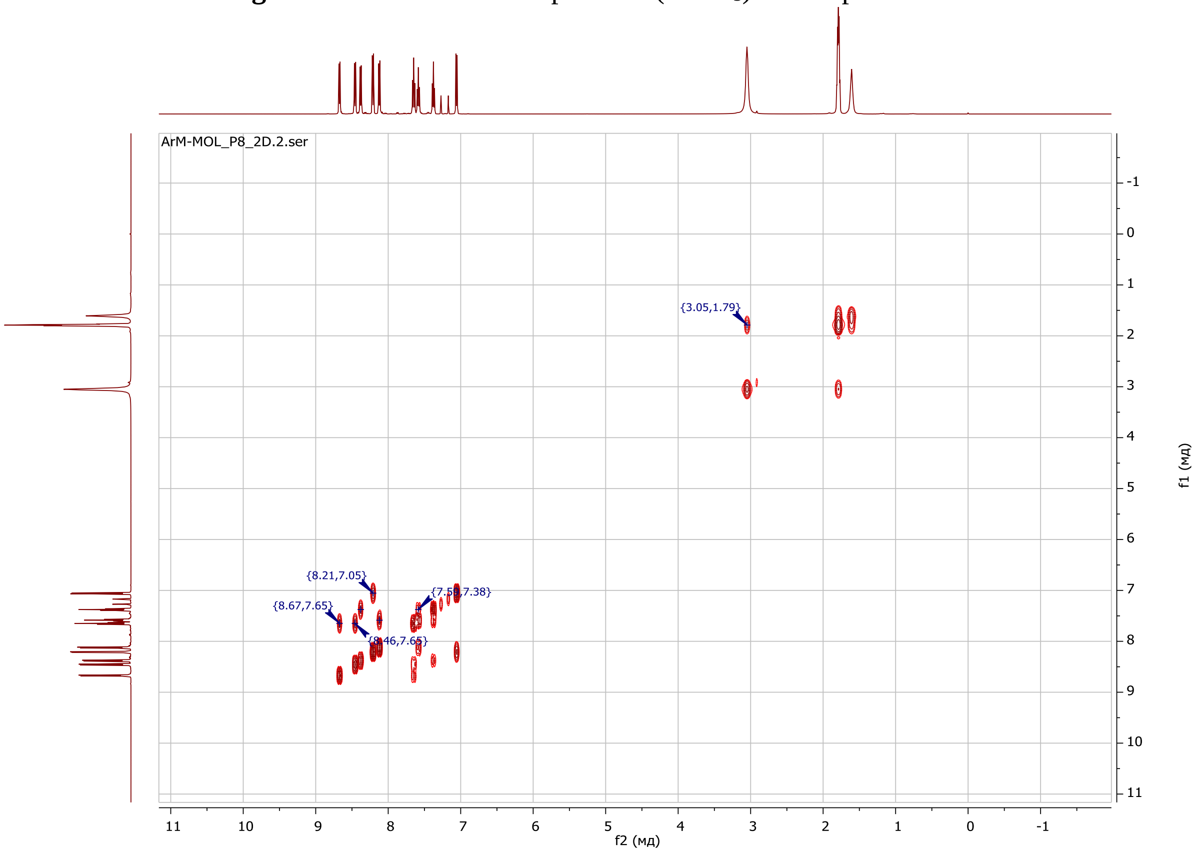

Figure S25. ^{1}H-^{1}H COSY spectrum ($CDCl_3$) of compound **6**.

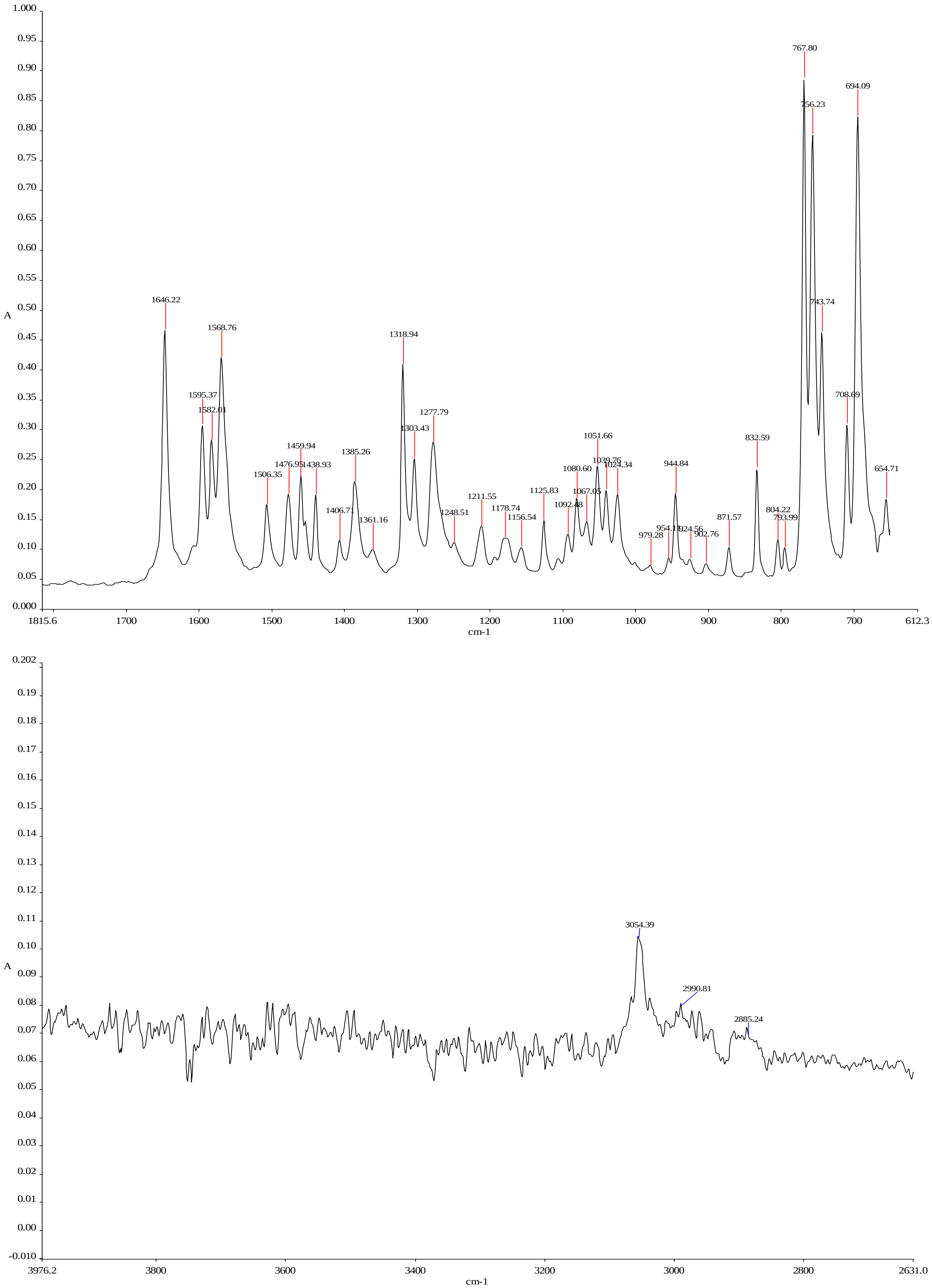

Figure S26. FTIR spectrum (neat) of compound **2.**

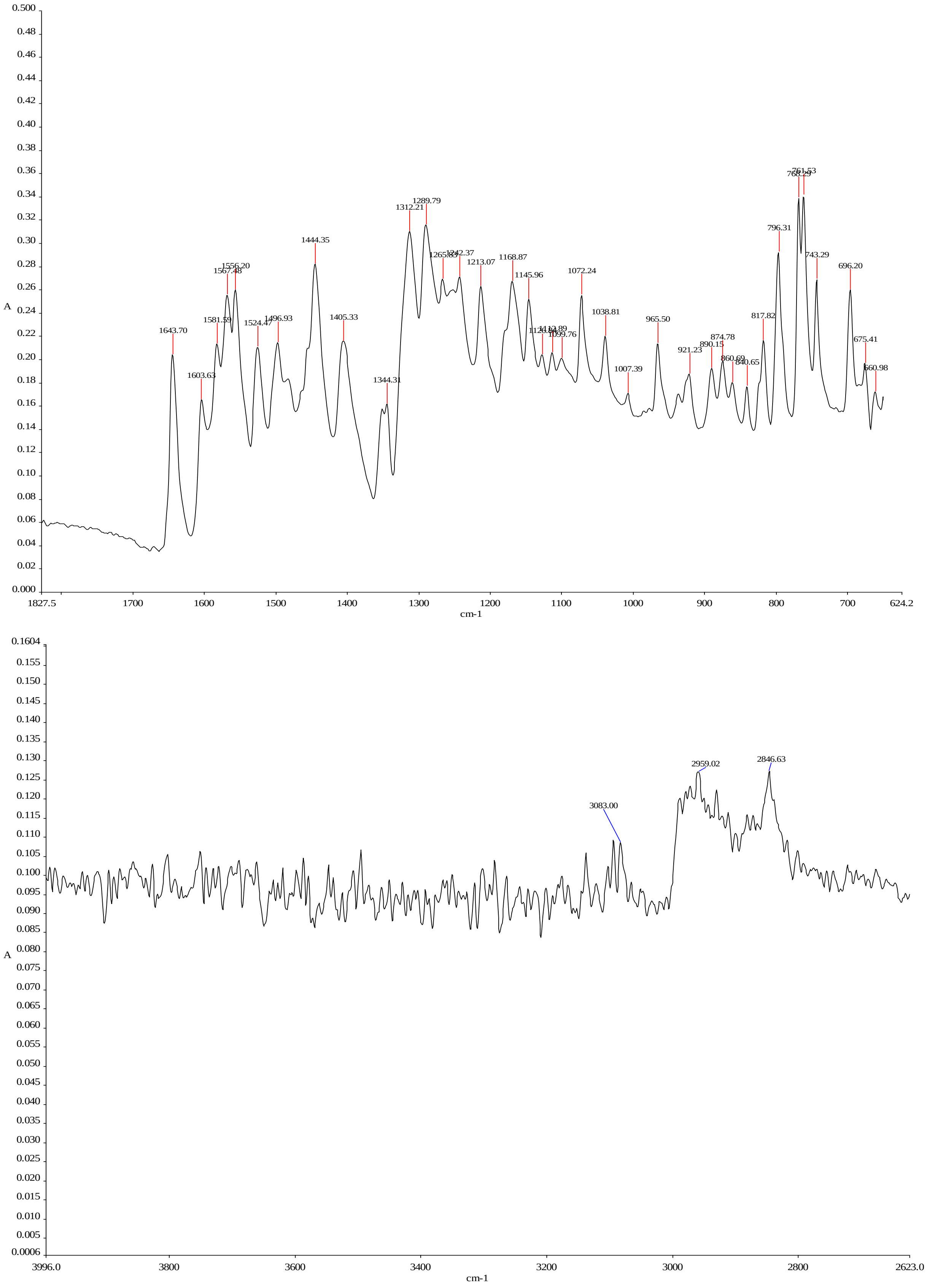

Figure S27. FTIR spectrum (neat) of compound **3.**

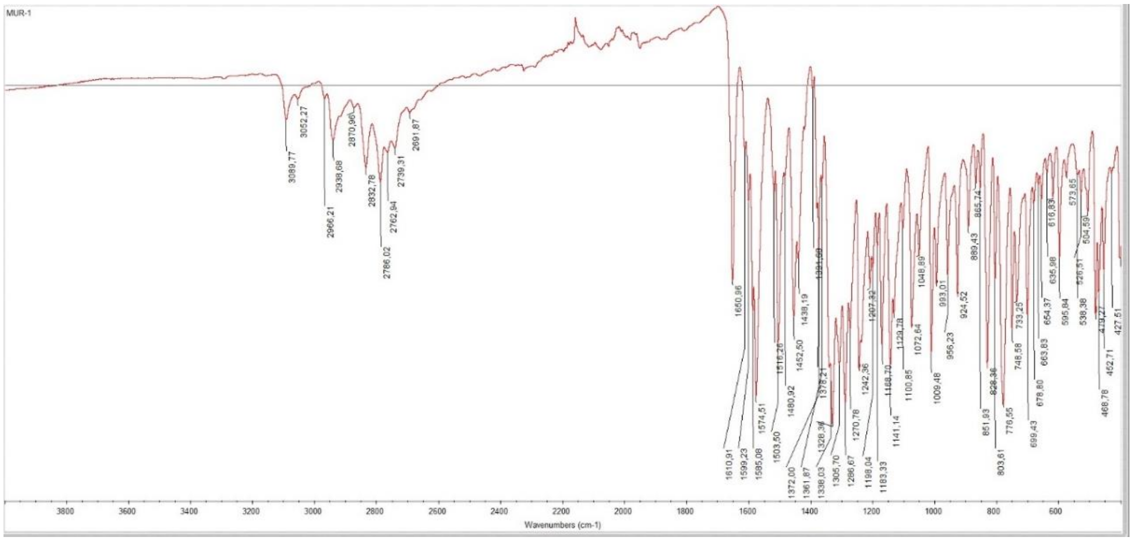

Figure S28. FTIR spectrum (neat) of compound **4.**

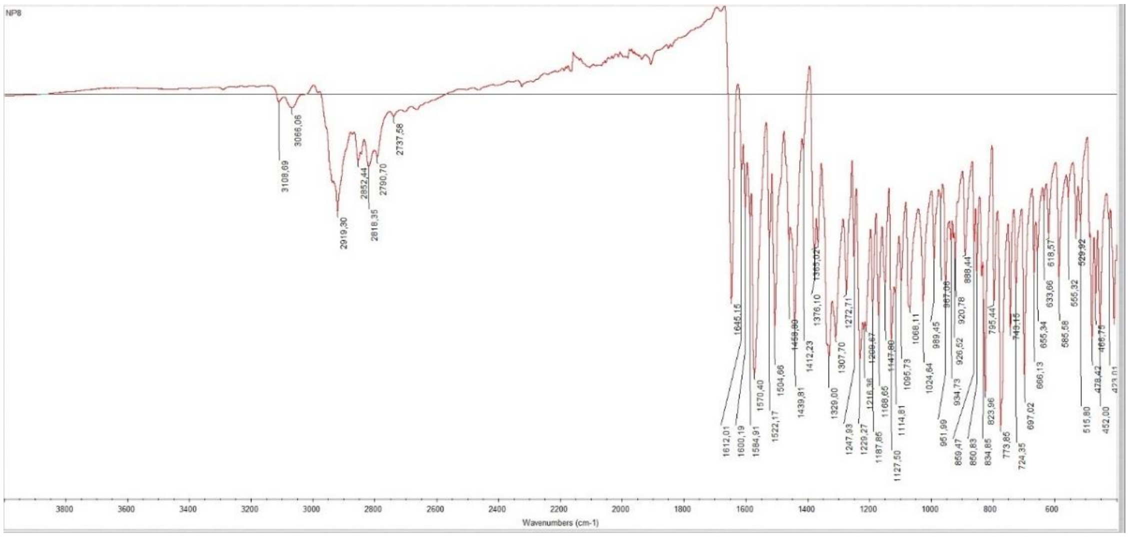

Figure S29. FTIR spectrum (neat) of compound **5.**

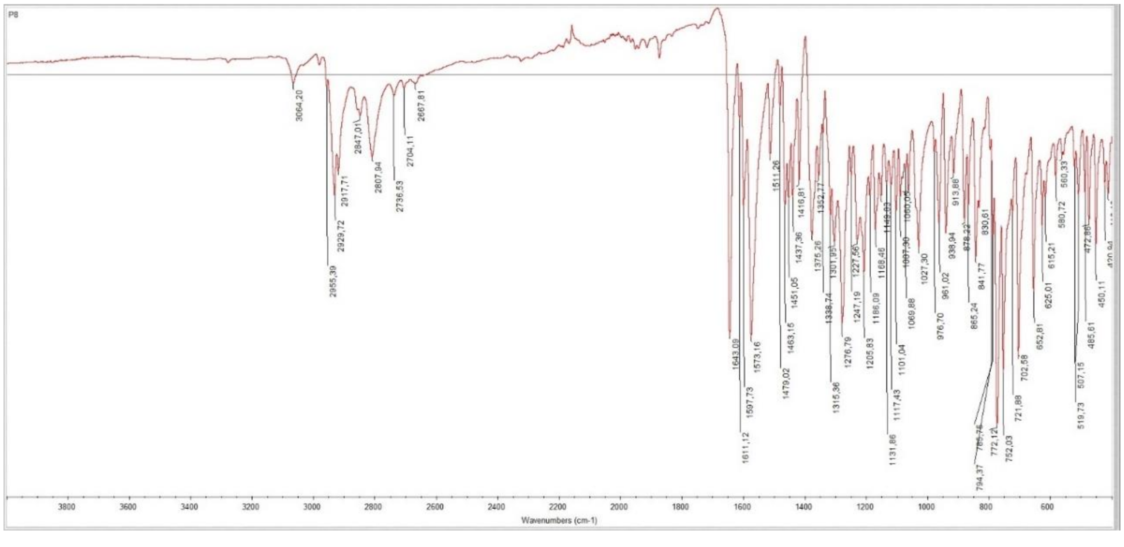

Figure S30. FTIR spectrum (neat) of compound **6.**

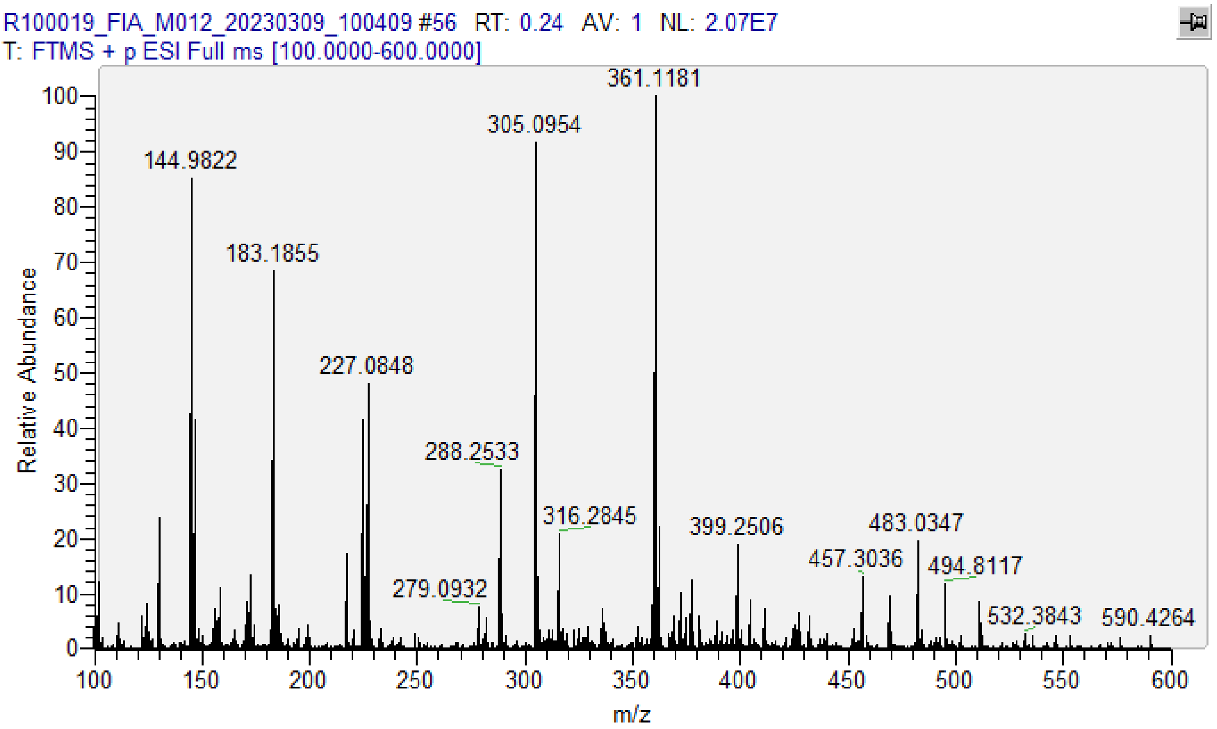

Figure S31. ESI-FTMS spectrum of compound **2.**

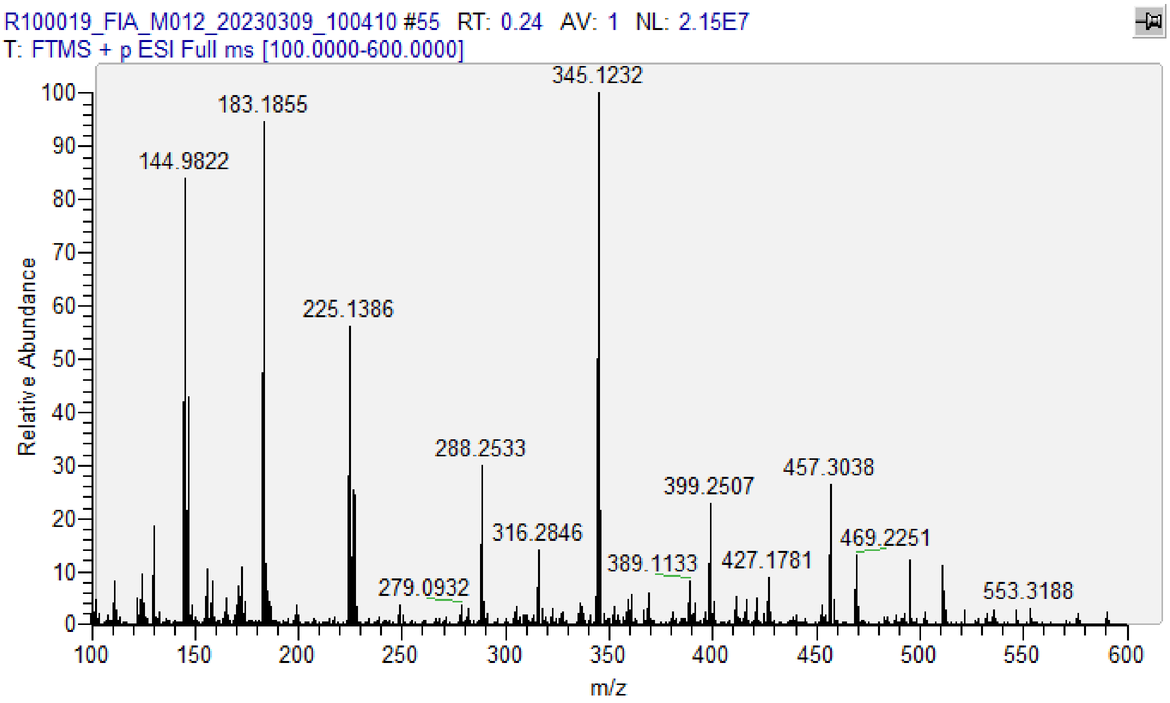

Figure S32. ESI-FTMS spectrum of compound **3.**

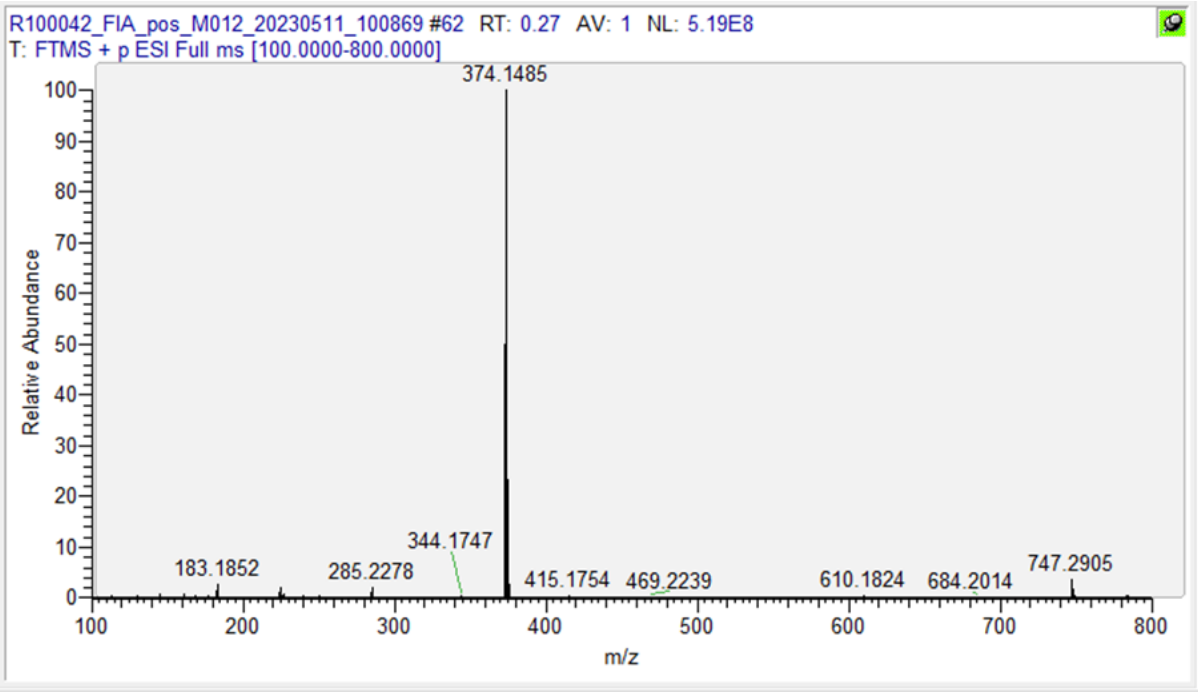

Figure S33. ESI-FTMS spectrum of compound **4.**

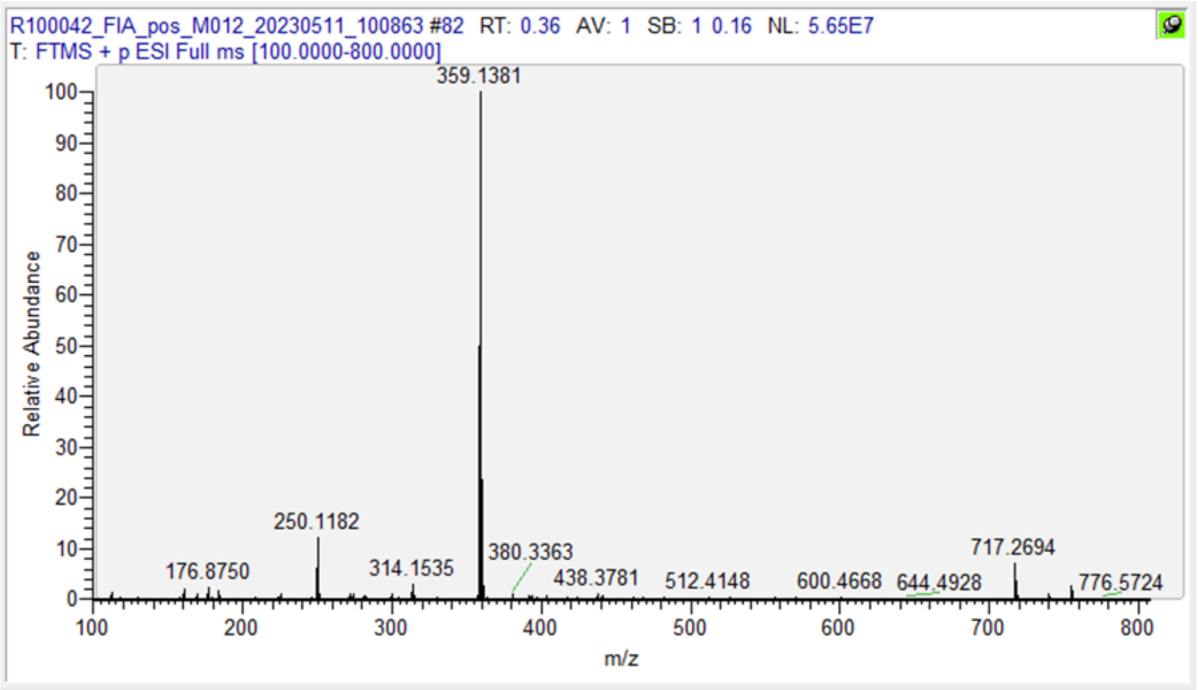

Figure S34. ESI-FTMS spectrum of compound **5.**

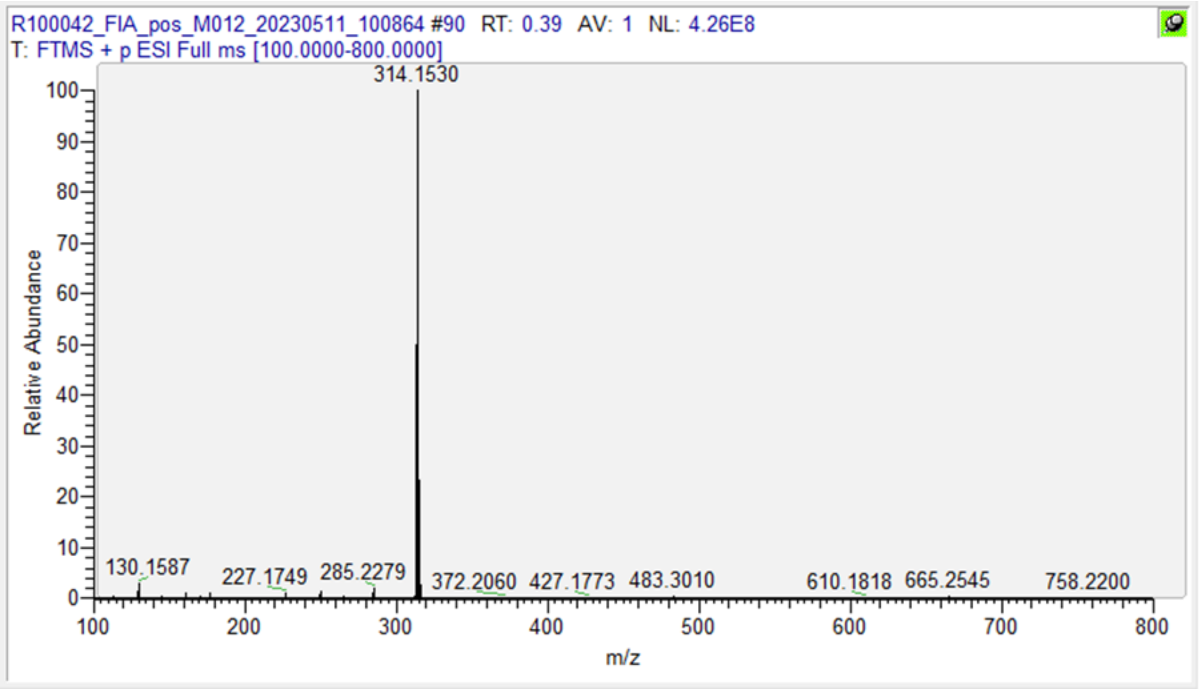

Figure S35. ESI-FTMS spectrum of compound **6.**

Table S1. Crystal data and structure refinement for compound **3**.

Empirical formula	$C_{21}H_{16}N_2O_3$
Formula weight	344.37
Temperature/K	140.0(1)
Crystal system	orthorhombic
Space group	*Pbca*
a/Å	14.4599(2)
b/Å	7.1736(2)
c/Å	29.6287(6)
α/°	90
β/°	90
γ/°	90
Volume/Å^3	3073.37(11)
Z	8
ρ_{calc}g/cm^3	1.4884
μ/mm^{-1}	0.822
F(000)	1440
Crystal size/mm^3	0.14 × 0.11 × 0.02
Radiation	Cu Kα (λ = 1.54184 Å)
2Θ max. for data collection/°	160
Index ranges	$-12 \leq h \leq 18$, $-9 \leq k \leq 7$, $-37 \leq l \leq 37$
Reflections collected	19757
Independent reflections	3342 [R_{int} = 0.0356, R_{sigma} = 0.0343]
Data/restraints/parameters	3342/0/251
Goodness-of-fit on F^2	1.037
Final R indexes [$I > 2\sigma(I)$]	R_1 = 0.0430, wR_2 = 0.1168
Final R indexes [all data]	R_1 = 0.0492, wR_2 = 0.1216
Largest diff. peak/hole / e Å^{-3}	0.28/-0.18

Table S2. Fractional Atomic Coordinates (×10[4]) and Equivalent Isotropic Displacement Parameters (Å[2]×10[3]) for compound **3**. U_{eq} is defined as 1/3 of of the trace of the orthogonalised U_{IJ} tensor.

Atom	*x*	*y*	*z*	*U*(eq)
C1	4179.1(9)	8040(2)	4323.6(5)	30.8(3)
C2	3991.9(9)	8060(2)	3864.4(5)	32.4(3)
C3	4630.3(9)	7429(2)	3543.7(5)	30.2(3)
C4	6292.1(10)	6490(2)	3419.5(5)	33.7(3)
C5	7129.4(10)	5870(2)	3581.2(5)	35.6(3)
C6	7261.1(9)	5651(2)	4042.7(5)	33.2(3)
C7	6764.9(9)	5976(2)	4830.9(5)	30.4(3)
C8	6248.2(10)	6666(2)	5604.4(5)	32.2(3)
C9	5589.6(10)	7284(2)	5901.9(5)	32.8(3)
C10	4725.8(10)	7915(2)	5756.5(5)	33.4(3)
C11	4536.4(10)	7947(2)	5302.4(5)	31.5(3)
C12	5197.2(9)	7358(2)	4981.5(5)	28.4(3)
C13	6055.3(9)	6695(2)	5142.7(5)	29.3(3)
C14	6575.7(9)	6156(2)	4342.9(5)	29.4(3)
C15	5703.1(9)	6823(2)	4186.3(5)	28.0(3)
C16	5012.4(9)	7402(2)	4497.6(5)	27.8(3)
C17	5540.2(9)	6898(2)	3707.7(5)	29.3(3)
N18	4386.4(8)	7352.8(19)	3096.6(4)	32.9(3)
C19	3449.6(11)	7948(3)	2963.4(6)	41.0(4)
C20	3353.3(11)	7324(3)	2476.1(5)	41.0(4)
C21	3918.8(10)	5541(3)	2467.6(5)	38.8(4)
C22	4753.7(10)	6031(2)	2758.7(5)	34.3(3)
O23	7479.7(7)	5252.1(17)	4972.4(3)	38.4(3)
N24	5789.9(9)	7295(2)	6382.0(4)	38.3(3)
O25	6541.1(8)	6693(2)	6511.7(4)	51.8(4)
O26	5198.0(9)	7903(2)	6643.2(4)	48.0(3)

Table S3. Anisotropic Displacement Parameters (Å^2×10^3) for compound **3**. The Anisotropic displacement factor exponent takes the form: $-2\pi^2[h^2a^{*2}U_{11}+2hka^*b^*U_{12}+\ldots]$.

Atom	U_{11}	U_{22}	U_{33}	U_{12}	U_{13}	U_{23}
C1	27.4(6)	39.1(8)	25.9(7)	-0.0(5)	1.1(5)	-1.8(6)
C2	27.0(6)	43.7(8)	26.4(7)	2.7(6)	-2.2(5)	-0.7(6)
C3	29.0(6)	37.5(7)	24.1(7)	-1.7(5)	-1.5(5)	1.4(5)
C4	30.5(7)	46.5(8)	24.0(7)	-1.3(6)	0.4(5)	0.9(6)
C5	27.9(6)	48.3(9)	30.5(7)	1.5(6)	3.6(5)	-1.8(6)
C6	25.3(6)	41.4(8)	32.9(7)	-0.3(6)	-2.0(5)	0.5(6)
C7	25.6(6)	35.6(7)	29.9(7)	-3.6(5)	-3.6(5)	1.2(5)
C8	29.9(6)	39.0(8)	27.6(7)	-5.5(6)	-4.0(5)	3.5(6)
C9	36.0(7)	39.6(8)	22.9(7)	-7.8(6)	-2.8(5)	1.5(6)
C10	33.5(7)	40.3(8)	26.3(7)	-4.2(6)	2.3(5)	-1.4(6)
C11	28.8(6)	39.4(8)	26.4(7)	-2.4(6)	-0.6(5)	-0.3(6)
C12	27.5(6)	32.2(7)	25.4(7)	-5.2(5)	-1.5(5)	0.3(5)
C13	28.0(6)	33.9(7)	26.0(7)	-4.6(5)	-2.5(5)	1.8(5)
C14	26.8(6)	34.4(7)	26.9(7)	-3.5(5)	-1.2(5)	1.1(5)
C15	26.4(6)	32.4(7)	25.3(7)	-3.4(5)	-1.1(5)	0.9(5)
C16	27.4(6)	33.0(7)	22.9(6)	-3.6(5)	-0.5(5)	0.4(5)
C17	27.5(6)	35.7(7)	24.8(7)	-1.8(5)	0.2(5)	0.3(5)

N18	28.6(5)	47.3(7)	22.9(6)	3.2(5)	-1.3(4)	-0.6(5)
C19	32.2(7)	62.1(11)	28.6(8)	8.1(7)	-6.4(6)	-4.5(7)
C20	33.5(7)	63.1(10)	26.5(7)	2.5(7)	-5.1(6)	-0.7(7)
C21	36.2(7)	55.0(9)	25.3(7)	-3.1(7)	-1.5(6)	-2.9(6)
C22	31.4(7)	48.2(9)	23.3(7)	0.9(6)	0.3(5)	-1.1(6)
O23	31.0(5)	52.9(7)	31.2(5)	4.5(5)	-5.8(4)	1.8(5)
N24	39.0(6)	50.9(8)	24.9(6)	-8.9(6)	-1.7(5)	2.4(5)
O25	38.8(6)	87.2(10)	29.5(6)	-5.6(6)	-8.7(5)	4.9(6)
O26	56.5(7)	62.4(8)	25.0(6)	1.7(6)	3.6(5)	-0.5(5)

Table S4. Bond Lengths for compound **3**.

Atom	Atom	Length/Å	Atom	Atom	Length/Å
C1	C2	1.387(2)	C9	N24	1.4517(19)
C1	C16	1.3882(19)	C10	C11	1.373(2)
C2	C3	1.400(2)	C11	C12	1.413(2)
C3	C17	1.4534(19)	C12	C13	1.4120(19)
C3	N18	1.3719(18)	C12	C16	1.4588(19)
C4	C5	1.376(2)	C14	C15	1.4270(19)
C4	C17	1.4132(19)	C15	C16	1.4216(19)
C5	C6	1.390(2)	C15	C17	1.4385(19)
C6	C14	1.380(2)	N18	C19	1.4741(18)
C7	C13	1.474(2)	N18	C22	1.478(2)
C7	C14	1.4771(19)	C19	C20	1.518(2)
C7	O23	1.2305(17)	C20	C21	1.518(2)
C8	C9	1.371(2)	C21	C22	1.525(2)
C8	C13	1.3963(19)	N24	O25	1.2306(18)
C9	C10	1.397(2)	N24	O26	1.2333(18)

Table S5. Bond Angles for compound **3**.

Atom	Atom	Atom	Angle/˚	Atom	Atom	Atom	Angle/˚
C16	C1	C2	122.48(13)	C7	C14	C6	118.36(12)
C3	C2	C1	122.25(13)	C15	C14	C6	120.89(13)
C17	C3	C2	117.05(13)	C15	C14	C7	120.75(12)
N18	C3	C2	119.92(13)	C16	C15	C14	120.55(12)
N18	C3	C17	123.03(13)	C17	C15	C14	118.55(12)
C17	C4	C5	122.25(14)	C17	C15	C16	120.90(12)
C6	C5	C4	119.99(13)	C12	C16	C1	122.08(13)
C14	C6	C5	120.40(13)	C15	C16	C1	117.72(12)
C14	C7	C13	117.01(12)	C15	C16	C12	120.18(12)
O23	C7	C13	121.26(13)	C4	C17	C3	123.29(13)
O23	C7	C14	121.73(13)	C15	C17	C3	119.19(12)
C13	C8	C9	119.08(13)	C15	C17	C4	117.51(12)
C10	C9	C8	121.84(14)	C19	N18	C3	118.89(12)
N24	C9	C8	119.55(13)	C22	N18	C3	125.97(12)
N24	C9	C10	118.61(13)	C22	N18	C19	109.56(12)
C11	C10	C9	119.08(14)	C20	C19	N18	104.68(13)
C12	C11	C10	121.30(13)	C21	C20	C19	102.41(13)
C13	C12	C11	117.87(13)	C22	C21	C20	102.87(13)
C16	C12	C11	122.08(12)	C21	C22	N18	104.28(12)
C16	C12	C13	120.05(12)	O25	N24	C9	118.69(13)
C8	C13	C7	118.01(12)	O26	N24	C9	118.58(13)
C12	C13	C7	121.17(12)	O26	N24	O25	122.73(13)
C12	C13	C8	120.80(13)				

Table S6. Hydrogen Atom Coordinates (Å×10^4) and Isotropic Displacement Parameters (Å^2×10^3) for compound **3**.

**Atom**	***x***	***y***	***z***	***U*(iso)**
H1	3719.7(9)	8479(2)	4526.6(5)	37.0(4)
H2	3410.6(9)	8518(2)	3764.1(5)	38.8(4)
H4	6216.8(10)	6648(2)	3103.3(5)	40.4(4)
H5	7617.5(10)	5593(2)	3377.4(5)	42.7(4)
H6	7826.4(9)	5150(2)	4152.2(5)	39.9(4)
H8	6827.7(10)	6223(2)	5711.0(5)	38.6(4)
H10	4275.5(10)	8316(2)	5968.8(5)	40.1(4)
H11	3949.4(10)	8373(2)	5202.1(5)	37.9(4)
H19a	3383(12)	9320(30)	2990(6)	35(4)
H19b	2976(15)	7300(30)	3149(8)	52(6)
H20a	2698.8(11)	7082(3)	2397.6(5)	49.2(4)
H20b	3608.8(11)	8265(3)	2265.9(5)	49.2(4)
H21a	3567.7(10)	4485(3)	2598.0(5)	46.6(4)
H21b	4108.6(10)	5218(3)	2156.2(5)	46.6(4)
H22a	5029(11)	4890(30)	2912(6)	34(4)
H22b	5253(12)	6630(30)	2569(6)	36(5)

Table S7. Crystal data and structure refinement for compound **5**.

Empirical formula	$2(C_{22}H_{18}N_2O_3)$
Formula weight	716.80
Temperature/K	150.0(1)
Crystal system	triclinic
Space group	$P\bar{1}$
a/Å	9.05934(12)
b/Å	12.12641(15)
c/Å	15.8953(2)
α/°	87.8669(10)
β/°	76.5700(11)
γ/°	86.6510(10)
Volume/Å^3	1695.05(4)
Z	2
ρ_{calc}g/cm^3	1.4043
μ/mm^{-1}	0.766
F(000)	752
Crystal size/mm^3	0.13 × 0.04 × 0.02
Radiation	Cu Kα (λ = 1.54184 Å)
2Θ range for data collection/°	160.0
Index ranges	$-11 \leq h \leq 11$, $-12 \leq k \leq 15$, $-20 \leq l \leq 20$
Reflections collected	30743
Independent reflections	7295 [R_{int} = 0.0367, R_{sigma} = 0.0345]
Data/restraints/parameters	7295/0/487
Goodness-of-fit on F^2	1.023
Final *R* indexes [$I > 2\sigma(I)$]	R_1 = 0.0417, wR_2 = 0.1181
Final *R* indexes [all data]	R_1 = 0.0475, wR_2 = 0.1233
Largest diff. peak/hole / e Å^{-3}	0.21/-0.22

Table S8. Fractional Atomic Coordinates (×10^4) and Equivalent Isotropic Displacement Parameters (Å^2×10^3) for compound **5**. U_{eq} is defined as 1/3 of of the trace of the orthogonalised U_{IJ} tensor.

**Atom**	***x***	***y***	***z***	***U*(eq)**
C1	7824.3(13)	10603.1(9)	3859.6(7)	26.9(2)
C2	7914.7(13)	11042.0(9)	3021.5(8)	27.7(2)
C3	7148.1(13)	10593.5(9)	2469.2(7)	25.3(2)
C4	5209.5(13)	9290.3(10)	2306.9(8)	27.5(2)
C5	4297.0(14)	8428.0(10)	2613.2(8)	30.6(2)
C6	4289.0(13)	7949.3(9)	3427.4(8)	29.2(2)
C7	5082.9(13)	7849.6(9)	4806.7(8)	26.2(2)
C8	5880.6(13)	7884.5(9)	6182.6(8)	27.6(2)
C9	6718.7(14)	8311.2(10)	6698.8(7)	29.0(2)
C10	7681.4(14)	9169.0(10)	6412.8(8)	30.6(2)
C11	7776.3(13)	9618.7(9)	5593.1(8)	28.1(2)
C12	6922.6(12)	9220.9(9)	5042.3(7)	23.8(2)
C13	5984.7(13)	8336.6(9)	5351.8(7)	24.9(2)
C14	5172.3(12)	8349.5(9)	3935.4(7)	24.7(2)
C15	6128.4(12)	9240.4(9)	3635.5(7)	23.0(2)
C16	6986.2(12)	9699.7(9)	4173.4(7)	23.2(2)
C17	6174.7(12)	9697.9(9)	2790.4(7)	24.3(2)
N18	7221.5(12)	11007.3(8)	1622.5(6)	28.2(2)
C19	7802.0(15)	12111.5(10)	1414.2(8)	33.6(3)
C20	7379.5(16)	12560.4(11)	588.4(8)	37.9(3)
C21	7970.2(17)	11787.4(12)	-163.1(9)	40.9(3)
C22	7446.1(16)	10624.9(12)	88.8(8)	39.1(3)
C23	7888.7(15)	10239.1(10)	921.3(8)	32.8(3)
O24	4303.2(11)	7058.7(7)	5073.0(6)	36.0(2)
N25	6590.4(13)	7853.7(9)	7589.0(7)	35.8(2)
O26	5872.5(13)	7022.6(8)	7789.2(6)	44.5(2)

O27	7192.2(17)	8334.1(11)	8063.2(7)	59.9(3)
C51	3147.0(13)	4774.3(9)	6148.0(7)	25.2(2)
C52	3384.0(13)	4661.3(9)	6986.6(7)	26.4(2)
C53	2629.3(12)	3897.5(9)	7578.6(7)	23.6(2)
C54	978.9(13)	2295.2(9)	7828.5(7)	25.8(2)
C55	13.8(14)	1616.9(9)	7566.7(8)	28.7(2)
C56	-306.9(13)	1788.0(9)	6754.3(8)	27.6(2)
C57	42.5(12)	2731.2(9)	5332.6(7)	25.1(2)
C58	556.7(13)	3692.1(10)	3911.4(7)	26.5(2)
C59	1310.1(13)	4463.1(10)	3338.8(7)	27.2(2)
C60	2341.3(13)	5138.5(10)	3565.9(7)	28.2(2)
C61	2625.9(13)	5031.3(9)	4378.8(7)	26.0(2)
C62	1881.8(12)	4251.4(8)	4991.1(7)	21.7(2)
C63	846.0(12)	3582.7(9)	4737.9(7)	22.8(2)
C64	369.1(12)	2611.0(9)	6196.6(7)	23.1(2)
C65	1385.8(11)	3316.3(8)	6447.0(7)	20.8(2)
C66	2155.1(12)	4130.1(9)	5863.2(7)	21.5(2)
C67	1643.3(12)	3177.5(9)	7296.4(7)	22.2(2)
N68	2827.7(11)	3764.1(8)	8422.7(6)	26.6(2)
C69	4211.9(14)	4170.8(11)	8597.7(8)	32.2(3)
C70	4496.3(15)	3618.5(12)	9423.2(8)	37.5(3)
C71	3141.4(17)	3816.9(13)	10185.5(8)	41.5(3)
C72	1691.0(15)	3466.0(12)	9968.7(8)	35.9(3)
C73	1488.5(14)	4026.9(10)	9126.8(7)	29.2(2)
O74	-854.3(11)	2144.9(8)	5108.0(6)	35.8(2)
N75	1016.4(12)	4590.7(10)	2464.2(7)	34.6(2)
O76	190.1(14)	3946.1(11)	2259.6(7)	52.3(3)
O77	1614.3(13)	5345.5(9)	1998.4(6)	44.9(2)

Table S9. Anisotropic Displacement Parameters (Å^2×10^3) for compound **5**. The Anisotropic displacement factor exponent takes the form: $-2\pi^2[h^2a^{*2}U_{11}+2hka^*b^*U_{12}+\ldots]$.

Atom	U_{11}	U_{22}	U_{33}	U_{12}	U_{13}	U_{23}
C1	28.4(5)	26.7(5)	26.6(6)	-5.9(4)	-7.6(4)	-0.2(4)
C2	29.8(5)	25.7(5)	28.0(6)	-8.7(4)	-6.1(4)	3.2(4)
C3	26.3(5)	24.5(5)	24.3(5)	-1.6(4)	-4.4(4)	1.1(4)
C4	29.0(5)	29.1(5)	24.3(5)	-1.8(4)	-5.3(4)	-3.6(4)
C5	31.9(6)	31.2(6)	30.6(6)	-6.8(5)	-8.8(5)	-6.9(5)
C6	29.9(6)	24.2(5)	32.9(6)	-7.6(4)	-4.3(5)	-3.3(4)
C7	26.7(5)	20.2(5)	29.5(6)	-3.2(4)	-1.3(4)	-0.9(4)
C8	30.3(6)	22.2(5)	26.6(6)	1.0(4)	0.1(4)	0.6(4)
C9	34.5(6)	27.3(5)	22.4(5)	3.7(4)	-2.5(4)	1.0(4)
C10	35.0(6)	29.4(6)	28.2(6)	0.3(5)	-9.5(5)	-2.0(4)
C11	30.5(5)	26.7(5)	27.2(6)	-4.5(4)	-5.9(4)	-0.2(4)
C12	24.0(5)	21.5(5)	24.1(5)	-0.1(4)	-2.0(4)	-1.8(4)
C13	26.1(5)	21.0(5)	24.9(5)	1.0(4)	-0.6(4)	-1.7(4)
C14	25.4(5)	20.2(5)	26.9(5)	-2.0(4)	-2.4(4)	-3.4(4)
C15	22.8(5)	20.8(5)	24.4(5)	-1.0(4)	-3.1(4)	-2.6(4)
C16	23.2(5)	21.5(5)	23.9(5)	-1.3(4)	-3.4(4)	-2.0(4)
C17	24.9(5)	23.3(5)	24.3(5)	-0.5(4)	-4.4(4)	-2.9(4)
N18	32.4(5)	27.6(5)	23.9(5)	-4.3(4)	-5.2(4)	2.6(4)
C19	39.4(6)	31.0(6)	30.9(6)	-8.1(5)	-8.8(5)	6.7(5)
C20	44.6(7)	36.5(6)	31.1(6)	-3.6(5)	-7.4(5)	10.1(5)
C21	42.8(7)	50.2(8)	26.5(6)	-0.6(6)	-3.8(5)	8.8(5)
C22	44.1(7)	46.8(7)	24.8(6)	0.5(6)	-5.3(5)	-1.1(5)
C23	35.7(6)	34.0(6)	26.4(6)	0.7(5)	-3.4(5)	0.2(5)
O24	42.2(5)	28.3(4)	37.1(5)	-14.9(4)	-6.3(4)	4.9(4)
N25	42.5(6)	31.4(5)	30.8(5)	2.6(4)	-4.5(4)	1.3(4)
O26	56.9(6)	40.1(5)	31.6(5)	-4.5(5)	-1.5(4)	9.0(4)

O27	90.7(9)	61.5(7)	35.4(6)	-18.7(6)	-28.5(6)	10.4(5)
C51	27.0(5)	25.0(5)	24.4(5)	-7.1(4)	-6.7(4)	3.5(4)
C52	28.1(5)	28.1(5)	25.4(5)	-7.9(4)	-9.8(4)	1.4(4)
C53	23.5(5)	26.7(5)	20.9(5)	-1.1(4)	-6.0(4)	0.2(4)
C54	27.9(5)	26.4(5)	21.9(5)	-1.5(4)	-3.4(4)	2.1(4)
C55	31.5(6)	24.7(5)	27.5(6)	-7.1(4)	-1.5(4)	3.3(4)
C56	26.9(5)	25.9(5)	29.8(6)	-8.2(4)	-4.3(4)	-1.3(4)
C57	23.9(5)	24.8(5)	27.4(5)	-3.2(4)	-6.2(4)	-4.5(4)
C58	25.6(5)	29.3(5)	25.5(5)	1.5(4)	-7.9(4)	-5.2(4)
C59	29.1(5)	32.4(6)	20.1(5)	5.6(4)	-7.1(4)	-2.8(4)
C60	29.8(5)	29.7(5)	23.3(5)	-1.3(4)	-3.4(4)	2.8(4)
C61	27.2(5)	27.6(5)	23.4(5)	-4.3(4)	-6.0(4)	1.1(4)
C62	21.2(5)	22.1(5)	21.2(5)	0.8(4)	-4.0(4)	-1.4(4)
C63	22.2(5)	23.7(5)	22.7(5)	1.2(4)	-5.5(4)	-3.5(4)
C64	22.3(5)	22.4(5)	24.4(5)	-2.3(4)	-4.2(4)	-2.1(4)
C65	19.9(5)	20.1(5)	21.9(5)	-0.9(4)	-3.6(4)	-1.5(4)
C66	21.7(5)	21.4(5)	21.5(5)	-1.0(4)	-5.4(4)	-0.3(4)
C67	21.6(5)	22.1(5)	22.1(5)	-0.8(4)	-3.6(4)	0.1(4)
N68	26.1(5)	35.0(5)	20.0(4)	-4.8(4)	-7.4(4)	0.8(4)
C69	30.6(6)	41.8(6)	27.1(6)	-8.7(5)	-11.9(5)	3.4(5)
C70	37.5(6)	48.9(7)	31.1(6)	-7.8(5)	-17.7(5)	5.7(5)
C71	50.7(8)	53.4(8)	24.6(6)	-9.8(6)	-16.3(6)	3.7(5)
C72	40.2(7)	45.1(7)	22.2(6)	-6.3(5)	-5.9(5)	1.3(5)
C73	31.0(6)	33.3(6)	23.0(5)	-1.8(4)	-5.7(4)	-1.1(4)
O74	39.0(5)	38.8(5)	34.4(5)	-16.8(4)	-14.6(4)	-1.0(4)
N75	34.9(5)	42.7(6)	25.8(5)	6.1(4)	-7.9(4)	-2.2(4)
O76	60.9(7)	70.1(7)	32.9(5)	-10.6(6)	-23.1(5)	-3.5(5)
O77	55.0(6)	53.0(6)	26.1(5)	2.7(5)	-10.4(4)	7.1(4)

Table S10. Bond Lengths for compound **5**.

Atom	Atom	Length/Å	Atom	Atom	Length/Å
C1	C2	1.4023(16)	C51	C52	1.3991(15)
C1	C16	1.3805(15)	C51	C66	1.3857(15)
C2	C3	1.3826(16)	C52	C53	1.3860(15)
C3	C17	1.4420(15)	C53	C67	1.4379(15)
C3	N18	1.4070(14)	C53	N68	1.3969(14)
C4	C5	1.3748(17)	C54	C55	1.3757(17)
C4	C17	1.4109(16)	C54	C67	1.4120(15)
C5	C6	1.3972(17)	C55	C56	1.3936(17)
C6	C14	1.3793(17)	C56	C64	1.3820(16)
C7	C13	1.4801(17)	C57	C63	1.4783(16)
C7	C14	1.4775(16)	C57	C64	1.4709(16)
C7	O24	1.2280(14)	C57	O74	1.2305(14)
C8	C9	1.3726(18)	C58	C59	1.3751(17)
C8	C13	1.3956(16)	C58	C63	1.3983(15)
C9	C10	1.3914(18)	C59	C60	1.3936(17)
C9	N25	1.4819(15)	C59	N75	1.4759(15)
C10	C11	1.3795(17)	C60	C61	1.3752(16)
C11	C12	1.4095(16)	C61	C62	1.4132(15)
C12	C13	1.4096(15)	C62	C63	1.4102(15)
C12	C16	1.4680(15)	C62	C66	1.4645(15)
C14	C15	1.4227(15)	C64	C65	1.4234(15)
C15	C16	1.4282(15)	C65	C66	1.4256(15)
C15	C17	1.4266(15)	C65	C67	1.4251(15)
N18	C19	1.4654(15)	N68	C69	1.4621(15)
N18	C23	1.4745(15)	N68	C73	1.4754(15)
C19	C20	1.5237(17)	C69	C70	1.5192(16)

C20	C21	1.523(2)	C70	C71	1.526(2)
C21	C22	1.523(2)	C71	C72	1.5208(19)
C22	C23	1.5195(17)	C72	C73	1.5228(16)
N25	O26	1.2233(15)	N75	O76	1.2156(16)
N25	O27	1.2107(17)	N75	O77	1.2250(16)

Table S11. Bond Angles for compound **5**.

**Atom**	**Atom**	**Atom**	**Angle/°**	**Atom**	**Atom**	**Atom**	**Angle/°**
C16	C1	C2	122.14(10)	C66	C51	C52	122.06(10)
C3	C2	C1	121.29(10)	C53	C52	C51	121.48(10)
C17	C3	C2	118.44(10)	C67	C53	C52	118.19(10)
N18	C3	C2	122.83(10)	N68	C53	C52	123.22(10)
N18	C3	C17	118.66(10)	N68	C53	C67	118.54(9)
C17	C4	C5	121.46(11)	C67	C54	C55	121.50(10)
C6	C5	C4	120.03(11)	C56	C55	C54	119.73(10)
C14	C6	C5	120.40(10)	C64	C56	C55	120.78(10)
C14	C7	C13	117.26(10)	C64	C57	C63	117.29(9)
O24	C7	C13	120.78(11)	O74	C57	C63	121.04(10)
O24	C7	C14	121.96(11)	O74	C57	C64	121.68(10)
C13	C8	C9	118.86(11)	C63	C58	C59	118.88(11)
C10	C9	C8	121.89(11)	C60	C59	C58	121.57(10)
N25	C9	C8	119.29(11)	N75	C59	C58	119.59(11)
N25	C9	C10	118.82(11)	N75	C59	C60	118.84(11)
C11	C10	C9	119.14(11)	C61	C60	C59	119.56(11)
C12	C11	C10	121.17(11)	C62	C61	C60	121.08(10)
C13	C12	C11	117.79(10)	C63	C62	C61	117.73(10)
C16	C12	C11	122.08(10)	C66	C62	C61	122.05(10)

C16	C12	C13	120.13(10)	C66	C62	C63	120.22(10)
C8	C13	C7	117.60(10)	C58	C63	C57	117.62(10)
C12	C13	C7	121.27(10)	C62	C63	C57	121.20(10)
C12	C13	C8	121.12(11)	C62	C63	C58	121.17(10)
C7	C14	C6	118.67(10)	C57	C64	C56	118.80(10)
C15	C14	C6	120.82(11)	C65	C64	C56	120.65(10)
C15	C14	C7	120.51(10)	C65	C64	C57	120.54(10)
C16	C15	C14	121.28(10)	C66	C65	C64	121.35(10)
C17	C15	C14	118.49(10)	C67	C65	C64	118.31(10)
C17	C15	C16	120.20(10)	C67	C65	C66	120.34(9)
C12	C16	C1	122.23(10)	C62	C66	C51	122.55(10)
C15	C16	C1	118.26(10)	C65	C66	C51	118.09(10)
C15	C16	C12	119.50(10)	C65	C66	C62	119.36(9)
C4	C17	C3	121.80(10)	C54	C67	C53	121.37(10)
C15	C17	C3	119.46(10)	C65	C67	C53	119.71(9)
C15	C17	C4	118.69(10)	C65	C67	C54	118.85(10)
C19	N18	C3	116.82(10)	C69	N68	C53	117.32(9)
C23	N18	C3	115.91(9)	C73	N68	C53	116.66(9)
C23	N18	C19	110.84(10)	C73	N68	C69	111.57(9)
C20	C19	N18	109.51(10)	C70	C69	N68	109.25(10)
C21	C20	C19	111.64(11)	C71	C70	C69	111.35(11)
C22	C21	C20	110.19(11)	C72	C71	C70	110.46(11)
C23	C22	C21	110.06(11)	C73	C72	C71	110.22(11)
C22	C23	N18	110.36(10)	C72	C73	N68	109.99(10)
O26	N25	C9	117.28(11)	O76	N75	C59	117.77(11)
O27	N25	C9	117.64(11)	O77	N75	C59	117.58(11)
O27	N25	O26	125.07(12)	O77	N75	O76	124.65(11)

Table S12. Hydrogen Atom Coordinates ($Å\times10^4$) and Isotropic Displacement Parameters ($Å^2\times10^3$) for compound **5**.

**Atom**	***x***	***y***	***z***	***U*(iso)**
H1	8357.2(13)	10938.3(9)	4223.3(7)	32.2(3)
H2	8514.3(13)	11659.5(9)	2829.5(8)	33.3(3)
H4	5189.7(13)	9619.0(10)	1757.4(8)	33.0(3)
H5	3671.5(14)	8157.4(10)	2271.1(8)	36.8(3)
H6	3671.4(13)	7344.6(9)	3632.4(8)	35.0(3)
H8	5240.7(13)	7291.6(9)	6387.4(8)	33.1(3)
H10	8265.8(14)	9441.6(10)	6777.4(8)	36.7(3)
H11	8428.6(13)	10206.8(9)	5396.3(8)	33.7(3)
H19a	7360.6(15)	12611.8(10)	1896.8(8)	40.3(3)
H19b	8920.5(15)	12072.7(10)	1333.8(8)	40.3(3)
H20a	6260.0(16)	12659.1(11)	690.2(8)	45.4(3)
H20b	7809.8(16)	13292.9(11)	436.4(8)	45.4(3)
H21a	7589.0(17)	12062.5(12)	-671.6(9)	49.0(4)
H21b	9094.4(17)	11771.8(12)	-323.4(9)	49.0(4)
H22a	6329.3(16)	10621.0(12)	170.3(8)	46.9(4)
H22b	7919.2(16)	10112.8(12)	-380.2(8)	46.9(4)
H23a	9009.3(15)	10199.5(10)	828.6(8)	39.3(3)
H23b	7524.9(15)	9490.0(10)	1087.5(8)	39.3(3)
H51	3682.8(13)	5309.6(9)	5761.8(7)	30.2(3)
H52	4077.9(13)	5118.4(9)	7154.0(7)	31.7(3)
H54	1203.4(13)	2167.0(9)	8379.8(7)	31.0(3)
H55	-432.3(14)	1034.3(9)	7938.1(8)	34.4(3)
H56	-998.3(13)	1333.5(9)	6581.8(8)	33.1(3)
H58	-148.5(13)	3241.4(10)	3748.2(7)	31.8(3)
H60	2844.5(13)	5669.6(10)	3161.8(7)	33.8(3)
H61	3334.3(13)	5489.0(9)	4531.3(7)	31.2(3)

H69a	5085.3(14)	4002.3(11)	8108.3(8)	38.6(3)
H69b	4099.4(14)	4982.2(11)	8662.8(8)	38.6(3)
H70a	4690.5(15)	2814.1(12)	9337.0(8)	45.0(3)
H70b	5411.7(15)	3913.7(12)	9554.6(8)	45.0(3)
H71a	3317.2(17)	3390.9(13)	10699.8(8)	49.8(4)
H71b	3032.6(17)	4610.6(13)	10325.5(8)	49.8(4)
H72a	1740.1(15)	2653.8(12)	9910.9(8)	43.1(3)
H72b	808.2(15)	3669.7(12)	10443.3(8)	43.1(3)
H73a	1358.5(14)	4836.8(10)	9198.7(7)	35.0(3)
H73b	565.0(14)	3770.3(10)	8978.4(7)	35.0(3)

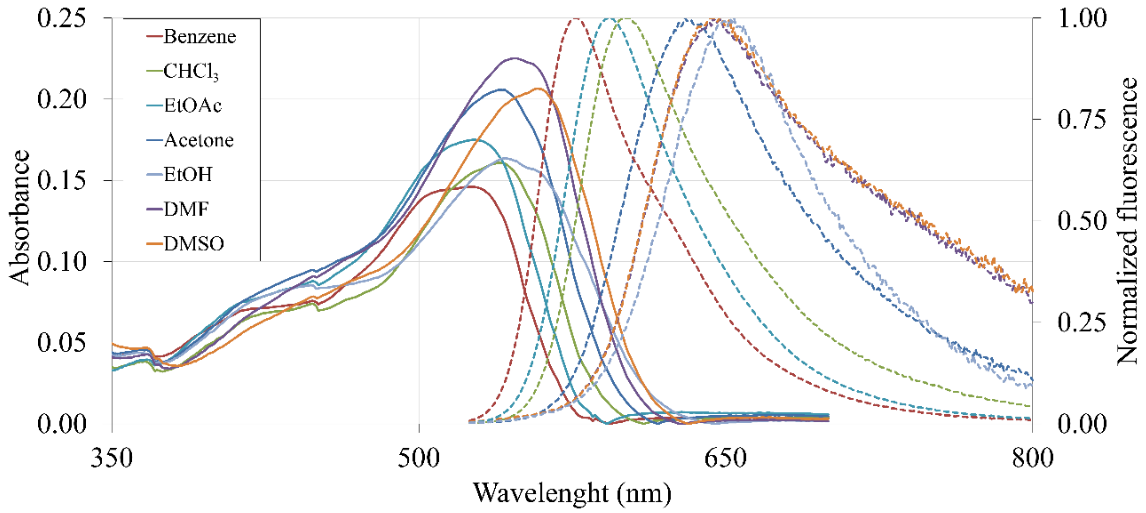

Figure S36. The absorption and emission spectra of compound **3** in various organic solvents.

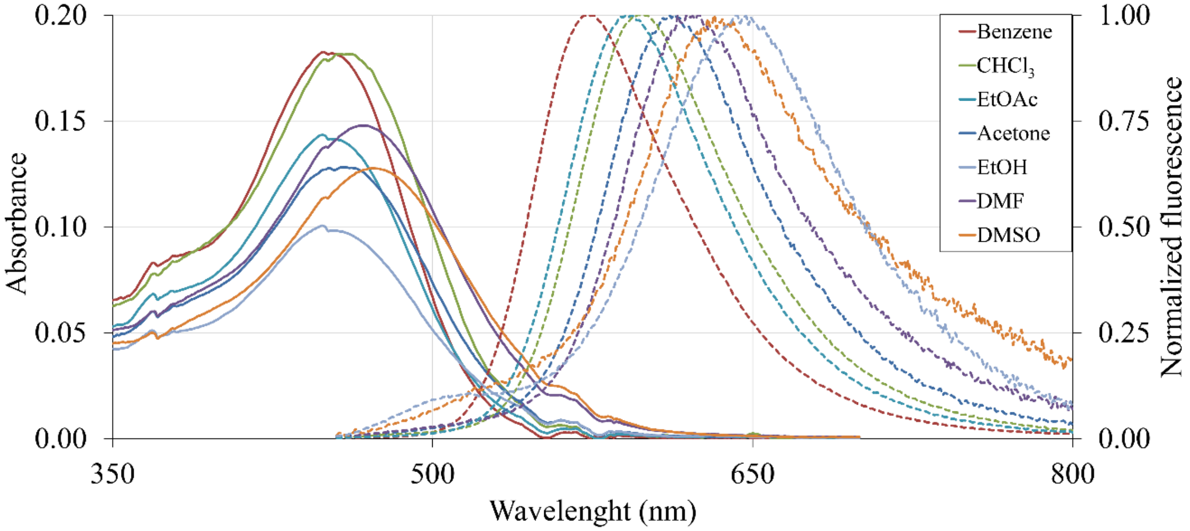

Figure S37. The absorption and emission spectra of compound **4** in various organic solvents.

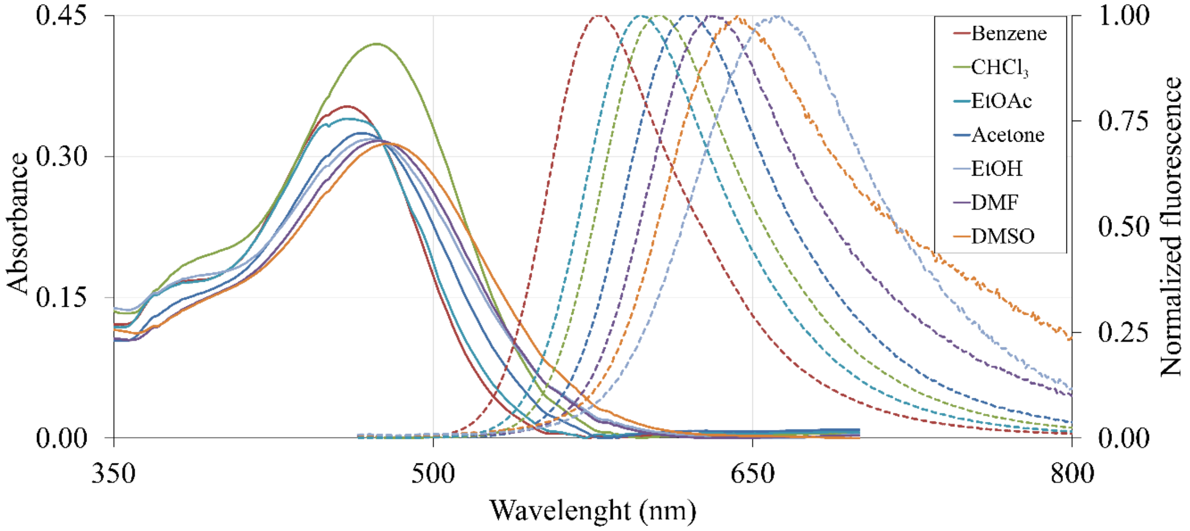

Figure S38. The absorption and emission spectra of compound **5** in various organic solvents.
